# Implicit emotion regulation in adolescent girls: An exploratory investigation of Hidden Markov Modeling and its neural correlates

**DOI:** 10.1371/journal.pone.0192318

**Published:** 2018-02-28

**Authors:** James S. Steele, Keith Bush, Zachary N. Stowe, George A. James, Sonet Smitherman, Clint D. Kilts, Josh Cisler

**Affiliations:** 1 Brain Imaging Research Center, Psychiatric Research Institute, University of Arkansas for Medical Sciences, Little Rock, Arkansas, United States of America; 2 Department of Computer Science, University of Arkansas at Little Rock, Little Rock, Arkansas, United States of America; 3 Women’s Mental Health Program, Psychiatric Research Institute, University of Arkansas for Medical Sciences, Little Rock, Arkansas, United States of America; University of Queensland, AUSTRALIA

## Abstract

Numerous data demonstrate that distracting emotional stimuli cause behavioral slowing (i.e. emotional conflict) and that behavior dynamically adapts to such distractors. However, the cognitive and neural mechanisms that mediate these behavioral findings are poorly understood. Several theoretical models have been developed that attempt to explain these phenomena, but these models have not been directly tested on human behavior nor compared. A potential tool to overcome this limitation is Hidden Markov Modeling (HMM), which is a computational approach to modeling indirectly observed systems. Here, we administered an emotional Stroop task to a sample of healthy adolescent girls (N = 24) during fMRI and used HMM to implement theoretical behavioral models. We then compared the model fits and tested for neural representations of the hidden states of the most supported model. We found that a modified variant of the model posited by Mathews et al. (1998) was most concordant with observed behavior and that brain activity was related to the model-based hidden states. Particularly, while the valences of the stimuli themselves were encoded primarily in the ventral visual cortex, the model-based detection of threatening targets was associated with increased activity in the bilateral anterior insula, while task effort (i.e. adaptation) was associated with reduction in the activity of these areas. These findings suggest that emotional target detection and adaptation are accomplished partly through increases and decreases, respectively, in the perceived immediate relevance of threatening cues and also demonstrate the efficacy of using HMM to apply theoretical models to human behavior.

## Introduction

Emotions are an important mechanism facilitating behavioral responses to salient and goal-related environmental cues [1], and the ability to regulate one’s emotions is an important determinant of health and well-being [2]. Emotions are multisystem phenomenon and regulation may be imposed at multiple points, including situation selection, attentional control, and response manipulation [3]. Tasks probing the automatic attentional control over salient stimuli (i.e. “implicit emotion regulation”), have been particularly successful probes for elucidating psychological and neurobiological aspects of emotion regulation [4, 5] and in characterizing abnormalities in the processes in patients with mood and anxiety disorders [6, 7]. However, while decades of research has shown that emotional stimuli alter behavioral responses, the exact mechanisms mediating the detection and regulation of conflicting or distracting emotional stimuli have not been clearly established. Delineating these mechanisms would have clear implications for understanding affective disorders characterized by deficits in emotion regulation ability. Towards this goal, we present here a novel empirical analysis of existing theoretical models of implicit emotion regulation.

Gross changes in emotion regulation strategy and aptitude take place over the course of development [8]. This is thought to be partially driven by imbalances in the developmental latency of subcortical brain regions compared to cortical regions, such that during adolescence subcortical brain regions are more fully developed and more sensitive to salient environmental cues, such as peer evaluations, and dominate behavior in the absence of mature cortical regulatory networks [9]. Also, adolescents are at a high risk for trauma exposure [10–12] and the development of mood and anxiety disorders [13, 14], both of which are related to abnormalities of emotion regulation [15, 16], making adolescence a critical period in which to study emotion regulation.

**Fig 1:**
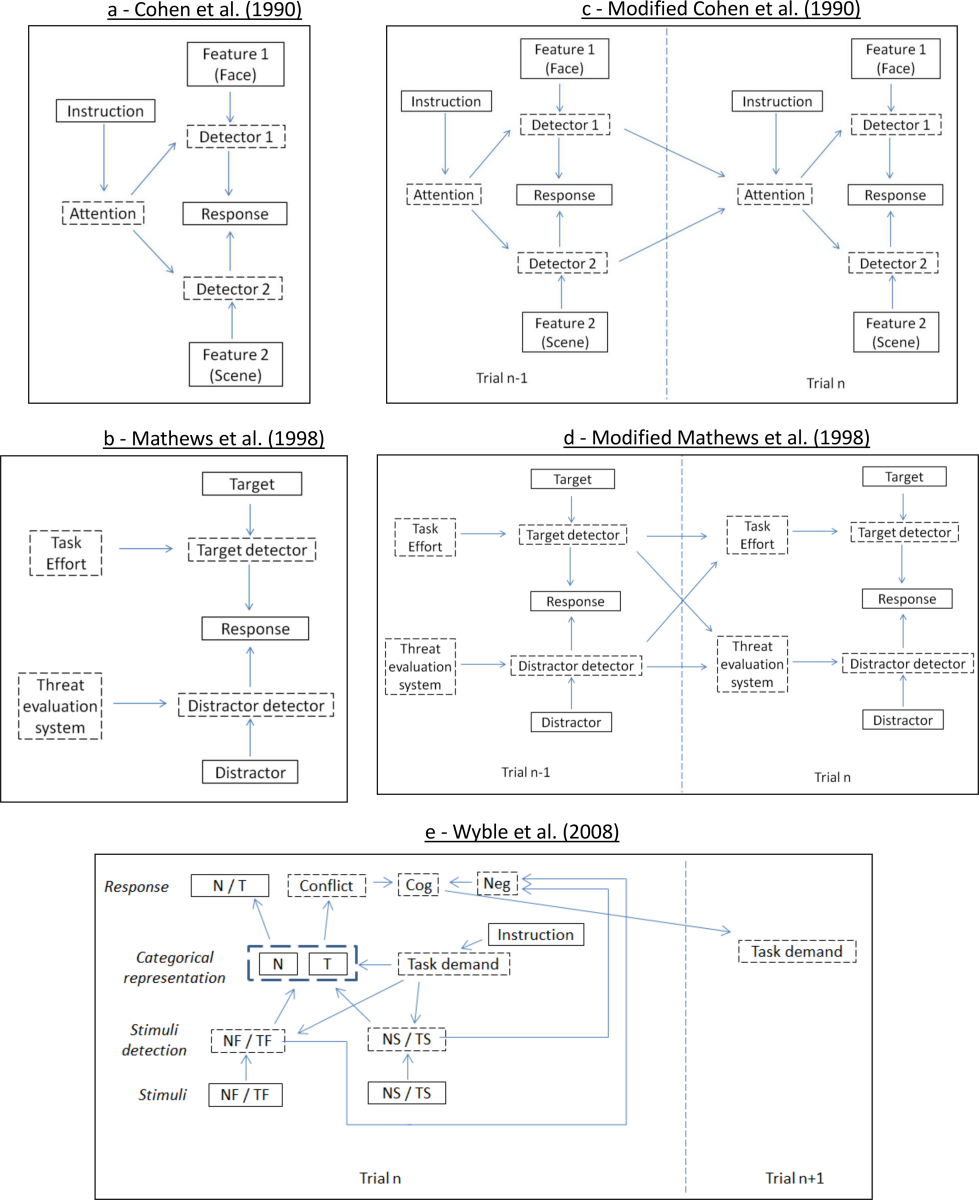
Schematic depiction of the 5 models implemented. The Cohen et al. (1990) and Mathews et al. (1998) models do not contain between-trial relationships (Fig 1A and 1B). The remaining model do, as depicted by the relationships between trial n-1 and trial n. (N = neutral, T = threat-related, F = face stimulus, S = scene stimulus, Cog = cognitive control, Neg = Negative emotion).

Emotional conflict processing tasks have drawn heavily from earlier studies of attentional control over nonemotional or “cognitive” distractors, which reliably demonstrated that naming ink color is slowed in the presence of a conflicting and task-incongruent color name [17, 18]. In order to provide a hypothetical model of the cognitive interference effect, Cohen et al. [19] postulated a framework that hypothesized the existence of a parallel distributed processing system (hereafter referred to as the “Cohen model”) (Fig 1A). According to this model, both pieces of information are simultaneously processed by distinct processing units, and the extent of interference is related to the automaticity and processing speed of each unit, which is modulated by conscious attentional control. Using simulations, it was demonstrated that the Cohen et al. model of parallel distributed processing accounts for the key findings related to cognitive interference tasks [19, 20].

Similarly, Mathews and Mackintosh [21] presented a related hypothetical model to explain findings specific to conflict tasks using *emotional* distractors (hereafter referred to as the “Mathews model”) (Fig 1B). Not directly influenced by the Cohen model, Mathews and Mackintosh posited a model based primarily on findings from implicit emotion identification and regulation tasks [21–23]. This model posits that attending to task relevant and irrelevant emotional stimuli is mediated by distinct processes and that anxiety specifically impairs the ability to ignore irrelevant threat-related information. It bears similarity to the Cohen model in that it is a parallel processing framework with separate processing nodes for the target and distractor stimuli. However, while the Cohen model specifies that attentional control alters processing of both the target and distractor stimuli, attentional control in the Mathews model only affects the processing of the target stimulus. Processing of the emotional distractor stimulus is modulated by a distinct system—the Threat Evaluation System (TES). This accurately models the sensitivity of attention to threatening stimuli, especially among anxious participants, who presumably have greater TES function, although simulations were not employed to test this model.

One shortcoming of both of these models is their inability to explain well-established dynamic changes in behavior that occur from trial to trial: namely, conflict adaptation or the “Gratton effect” [24, 25], and the “slow component” of cognitive [26] and emotional [27, 28] interference. The Gratton effect refers to the finding that the degree to which a conflicting distractor stimuli impairs performance is dependent on whether conflict was present in the previous trial (i.e. individuals adapt to the difficulty of the task). The slow interference component refers to the fact that block and event-related task designs produce different levels of performance bias, such that blocked presentations of emotional stimuli produce greater interference effects than a single emotional stimulus presentation. However, the Cohen model and Mathews model do not account for these dynamic effects without some modification to include between-trial dependencies. Botvinick et al. [29] applied these modifications to the Cohen model (hereafter referred to as the “modified Cohen model”) (Fig 1C) and found, using simulations, that the modified Cohen model was concordant with the Gratton effect and slow cognitive component. However, the authors did not test specifically for models that address the slow emotional interference effect. A recent study provides a more elaborate model shown via simulations to account for the slow emotional interference effect, as well as dynamic changes in cognitive control during implicit emotion regulation (hereafter referred to as the “Wyble model”) (Fig 1E) [30]. The Wyble model expands upon the previous models of parallel processing and incorporates features allowing for interaction between emotional and cognitive processes.

Neuroimaging has also proven to be a valuable tool in characterizing cognitive and emotional processes. Studies of the neural correlates of cognitive conflict processing have found that the dorsal anterior cingulate cortex (dACC) detects stimulus conflict and triggers the recruitment of the dorsolateral prefrontal cortex (dlPFC), which then enables cognitive control through the amplification of the neural response to the task-relevant feature [4, 31–33]. Botvinick et al. [29] demonstrated that activity of a conflict monitoring node implemented in the Cohen model of cognitive control parallel findings of dACC activity. Additionally, Wyble et al. [30] found that their model was consistent with dACC-induced activation of the dlPFC on the subsequent trial.

With respect to implicit emotion regulation, overlapping yet distinct neural systems are involved. The dACC is also responsive to emotional conflict, but adaptation occurs through inhibition of the amygdala by the pregenual ACC/ventromedial PFC (pgACC) [4, 5]. In addition, the ACC is highly interconnected with the anterior insular cortex forming the hubs of the “salience” network, which is thought be related to arousal and alertness [34, 35]. The anterior insula itself is activated by a broad array of tasks and is thought to be generally related to awareness [36]. However, none of the prior modeling studies have tested for concordance between model predicted implicit emotion regulation performance and neuroimaging findings.

Despite the insight into cognitive and emotional processes that these models have allowed and their success in reproducing human behavior, the models have only been indirectly tested on actual human data. That is, simulations have been done to test the ability of the models to recreate human behavior, but the models have not been directly fit to human data to test the degree to which the models explain observed human data. Similarly, neuroimaging studies have demonstrated that particular nodes (e.g., dACC, lateral PFC) are implicated in cognitive processes specified in the models (e.g., conflict detection, emotion regulation), but these studies only indirectly test the models through use of static contrast analyses instead of actually testing the neural correlates of the latent constructs specified in the models and derived through fitting the model to observed behavioral data. Thus, our theoretical models of how emotional conflict is detected and resolved have only been weakly tested in regards to both behavioral and neuroimaging data.

One potential approach to directly testing these theoretical models and identifying the cognitive and neural mechanisms mediating emotional conflict detection and resolution comes from the field of computational modeling. Computational models have been shown to explain reinforcement learning behavior [37] and an adaptation of the Rescorla-Wagner model to emotion regulation was recently proposed [38]. Another form of computational model, which is a nodal and probability-based model, is the hidden Markov model (HMM). HMM’s are a class of dynamic bayesian network and are a flexible analytical approach to implementing temporal models of indirectly observed “hidden” states, such as the stimulus processors or the threat evaluation system posited in the models described above. HMM’s represent models as a system of discrete nodes connected by conditional probabilities. They have been used extensively in the construction of algorithms for speech recognition [39, 40] and more recently to integrate fMRI activity with measures of cognitive performance in order to obtain more accurate assessments of hidden cognitive states [41–43], as well as to directly compare theoretical models of cognitive processing using EEG data [44].

Here, as part of a larger study of the effects of assault victimization on development and psychopathology, we recruited a cohort of healthy control adolescent girls and administered a variant of the emotional Stroop task. We then use Hidden Markov Modeling to test the fit of the above models of cognitive and emotional control to behavior on the task. The models are then compared in their fits to the data and the best fitting model is used in an exploratory analysis of the neural correlates of the model. We hypothesize that the model-based subprocesses of emotional conflict processing will be associated with similar neural mechanisms as the contrast-based measures of neural activity (dlPFC, pgACC, dACC, and anterior insula). However, using this modeling-based approach to delineate the subprocesses of emotional conflict processing allows a greater degree of functional separation in the involvement of the implicated brain regions. While the posited models differ in their description of the behavioral phenomena, we hypothesize that activity of the dACC and anterior insula will be related to stimulus processing while activity in the dlPFC and pgACC will be associated with attentional control.

## Methods

### 2.1 Participant recruitment and screening

The study was conducted in accordance with the Declaration of Helsinki and approved by the University of Arkansas for Medical Sciences Institutional Review Board. As part of larger study of the effect of trauma exposure on emotion regulation, 32 non-traumatized adolescent girls, 12–16 years of age, were recruited from the local area via newspaper advertisements and flyers. After the nature of the study had been fully explained to the participant and the participant’s parent or legal guardian and they both agreed to participation, written informed consent was obtained from the parent/legal guardian and written assent was obtained from the participant.

In addition to screening for trauma exposure with the trauma assessment portion of the National Survey of Adolescents [13, 14], participants were also screened for current symptoms of mental illness, given that mental illness is associated with abnormalities in the neural and behavioral processing of emotional stimuli [6, 7, 15]. This was done using either the K-SADS [45] (n = 21) or the Mini International Neuropsychiatric Interview (MINI) (n = 11).

Of the 32 non-traumatized adolescents assessed 2 had current diagnoses mental illnesses, 5 had excessive head-motion during the fMRI scan, and 1 was scanned with incorrect imaging parameters. All remaining subjects performed the task adequately (>75% accuracy). Therefore, all subsequent analyses were conducted on a final sample of 24 healthy adolescent girls.

### 2.2 Implicit emotion regulation task

Participants underwent fMRI while being administered a variant of the emotional Stroop task. In this task participants were simultaneously presented with two images, one superimposed upon the other. The background image depicted a nature scene, either threat-related (e.g. snake or spider) or neutral (e.g. foliage or rocks), while the superimposed foreground image was of a person’s face making either a threat-related or neutral expression. The valences of the two images were factorially manipulated (threatening/neutral background x threatening/neutral face) and uncorrelated. The participants were instructed either to attend to the scene or to the face and make a button press to indicate whether that stimulus (the ‘target’ stimulus) was threat-related or neutral. Responses were made using the first two buttons of the Current Designs 4 Button Curved (HHSC-1x4-CR). Neurobehavioral Systems Presentation software was used for stimulus presentation and timing recording.

The task was administered using a mixed block- event-related design in which each run was comprised of two blocks, one block in which the participant was instructed to attend to the face and the other to the scene. Within the blocks an event-related design was used with a jittered interstimulus interval of 3–7 seconds. The sequence of trial types (feature valences) was psuedo-randomized and identical across participants. In order to reduce contributions of lower-level priming effects, stimulus features were not repeated on successive trials. Three runs of 64 trials each (192 total trials) were administered with each run lasting approximately 8 minutes.

### 2.3 Task validation

In order to test the validity of our task as a probe of implicit emotion regulation we performed standard contrast-based analyses of task performance. Each subject’s reaction time bias was calculated by taking the difference between the median reaction times of the task conditions comprising the contrast, using only trials in which the participant responded correctly. Then, a robust one-tailed t-test was used to test for consistent effects of the task manipulation across the group. Specifically, we tested for: a main effect of conflict, a specific effect of conflict with a threatening stimulus as the distractor, a specific effect of conflict with a threatening stimulus as the target, an effect of adaptation to conflict, and the effect of non-conflict threat-related stimuli on subsequent responses to non-conflict neutral stimuli (slow emotional interference).

### 2.4 fMRI image aquisition and preprocessing

A Philips 3T Achieva X-series MRI system with an 8-channel head coil (Philips Healthcare, USA) was used to acquire imaging data. Anatomic images were acquired with an MPRAGE sequence (matrix = 192x192, 160 sagittal slices, TR/TE/FA = 7.5/3.7/9°, final resolution = 1x1x1mm3 resolution). Echo planar imaging sequences were used to collect the functional images using the following sequence parameters: TR/TE/FA = 2000ms/30ms/90°, FOV = 240x240mm, matrix = 80x80, 37 oblique slices (parallel to AC-PC plane to minimize OFC sinal artifact), slice thickness = 3 mm, final resolution 3x3x3 mm3.

Prior to analysis, standard image preprocessing was performed with AFNI [46]. The following preprocessing steps were applied in a specified order: despiking, slice timing correction, deobliquing, motion correction using rigid body alignment, alignment to participant’s normalized anatomical images. The MNI template at 3mm resolution was used for subject coregistration. Fluctuations in white matter voxels and CSF were then regressed out of the time courses from grey matter voxels to correct for non-neuronal artifacts. This image segmentation used restricted maximum likelihood to account for autocorrelation following segmentation [47]. Next, images were spatially smoothed with a 5.0 mm FWHM Gaussian kernel and scaled to percent signal change. Finally, to further correct for residual motion artifacts that standard motion correction does not remove, independent component analyses (ICA) were performed separately on each run of each participant to identify and remove artifact components [48–50].

### 2.5 Model implementation

A Hidden Markov Model is comprised of nodes and the conditional probabilities connecting the nodes. Although the above simulation studies [19, 29, 30] used activation simulations in which nodes could take on a range of continuous values, the models described can be tested using a binary classification of the hidden nodes (e.g. the threat evaluation system of the Mathews et al. model is either enhanced or inhibited on any given trial). Modeling the nodes in this way reduces the complexity of the models, and future studies can investigate the advantage gained by using continuous hidden nodes, which necessitates the use more complex modeling techniques, such as Kalman filtering. Nodes are linked to one another via conditional probability tables (CPT’s). These CPTs describe the interdependencies between nodes probabilistically. For example, consider a simple system of two connected binary nodes, A and B, in which B is dependent on A. The CPT describing their relationship is expressed as a 2x2 table with the entries in each row summing to 1. The first row describes the probability of B being either 0 or 1, given that A is 0, and the second row describes the probability that B is 0 or 1, given that A is 1. Each CPT requires x*(y-1) parameters to describe, where x is the number of rows (the number of states A may take on) and y is the number of columns (the number of states B may take on).

The models were implemented as depicted in Fig 1. The observed nodes (the stimuli and the responses) are marked by solid boxes, while the hidden nodes are marked by dashed boxes. All nodes, save the response node, were modeled as binary (enhanced/suppressed) values. In order to discretize the reaction times to make them amenable for use in the HMM’s, for each individual a median split was applied to their correct reaction times, such that all correct responses faster than their median correct reaction time were discretized to a single value ‘fast’, while all correct response times slower were discretized to ‘slow’. This discretization sacrifices power for reductions in model complexity while retaining a linear interpretation of reaction time. Incorrect and missing responses were discretized to a single value, ‘error’. Therefore, on any given trial 3 responses were possible: fast, slow, or error. These 3 values allow for the detection of task effects on response rates, while introducing the least amount of complexity. Future studies could test whether the finer-grained or continuous characterization of participant responses aids in model performance. Model implementation and parameter fitting was performed using the Bayes Net Toolbox for Matlab [51].

#### 2.5.1 Cohen model implementation

The Cohen model (Fig 1A) is a general model of parallel stimulus processing not specific to emotional conflict [19]. According to this model there are 3 hidden nodes that link the stimuli and instructions to the response. Two of the nodes act as detectors of the stimulus features, in which the processors are dedicated to their specific feature, regardless of whether that feature is the target or the distractor. In the emotional Stroop task used here, the features comprising the stimuli are a threat-related or neutral facial expression and a threatening or neutral nature scene. According to the Cohen model, there exists a detector (“detector 1”) for the valence of the facial expression (“feature 1”), and a detector (“detector 2”) for the valence of the scene (“feature 2”). These detectors are biased toward the detection of their respective feature by the “attention” node, which sensitizes the appropriate detector based on the current task demands (i.e. what the participant has been instructed to attend to). The detectors then interact to produce the observed response.

#### 2.5.2 Modified Cohen model implementation

As described above, one limitation of the Cohen model is that it does not capture dynamic temporal effects across trials (the Gratton effect and the slow component of interference). To account for these effects, Botnivick et al. applied a conflict monitoring feedback node which connects competition between response tendencies to the task demand node of the subsequent trial [29]. Here, we apply this same modification and test it separately from the standard Cohen model (Fig 1B).

#### 2.5.3 Mathews model implementation

The model of implicit emotion regulation described by Mathews et al. (Fig 1C) hypothesizes the existence of 4 hidden nodes that act as intermediates between the features of the stimulus presented and the response observed [21]. These nodes are the “target detector”, “distractor detector”, “task effort”, and “threat evaluation” nodes. The target detector and distractor detector are analogous to the detector of the Cohen model, except in this model instead of being specific to one of the stimulus features they are specific to whatever is the target or distractor on the current trial. Therefore, this model distinguishes between the detection of threat based on whether the participant intends (the target) or does not intend (the distractor) to detect it. The task effort node alters the target detector’s response to the target feature of the stimulus, such that high task effort causes the target detector to be more sensitive to the valence of the target, regardless of whether the target is threat-related or neutral. The threat evaluation node alters the response of the distractor detector to the distractor. High threat evaluation causes the distractor detector to be more likely to detect a threat-related distractor. The target detector and distractor detector then interact, producing a final stimulus representation that governs the response. Mathews et al. also posited that the target detector and distractor detector interact with one another to produce a final stimulus representation [21]. However, this interaction is not tenable under an HMM framework because it introduces a cycle between two hidden states leading to an unstable model. Therefore, we modeled this interaction as occurring at the level of response generation, which still allows these two nodes to interact but does not introduce a cycle into the model.

#### 2.5.4 Modified Mathews model implementation

In order to model the Gratton effect and the slow component of emotional and cognitive interference, we separately tested a model in which we applied a modification similar to the one applied by Botvinick et al.[29]. This modification allowed the target detector and distractor detector from the previous trial to influence the task effort and threat evaluation of the current trial (Fig 1D). This modification enables the presence of dynamic changes in cognitive and emotional control that are not present in the Mathews model.

#### 2.5.5 Wyble model implementation

The model of implicit emotion regulation posited by Wyble et al. (Fig 1E) is the most complex of the three models, requiring 7 hidden nodes to be implemented in the HMM framework [30]. Similar to the Cohen model, the Wyble model also has feature specific detectors which are biased by a task demand node. However, the detectors, rather than directly converging on the response layer, converge on an intermediate “categorical” layer, which is also biased by task demand toward processing of the task relevant feature. Output from the categorical layer then produces the response tendencies. If these response tendencies conflict, this conflict triggers cognitive control on the next trial, via amplification of the task demand node. Additionally, to explain the slow emotional interference effect, the detection of negative information for the detectors causes reductions in the recruitment of cognitive control. Because we observe responses subsequent to the resolution of conflicting response tendencies, in order to implement this model using the HMM framework we introduced a conflict node which represents the presence of conflict at the categorical level, rather than the response level.

### 2.6 Model fitting

To find the set of parameters that provided the best fit of the data to a given model, we applied the Baum-Welch algorithm [52], which is an iterative expectation maximization procedure. The Baum-Welch algorithm starts with an initial set of parameter estimates and makes iterative adjustments to them, maximizing the probability of the observed evidence given the model (i.e. the negative log-likelihood is maximized) for each iteration, until further adjustment does not result in better fit of the model to the evidence. The evidence supplied for the model fitting are the stimuli, response, and instruction (if applicable) for each trial. Model fitting was performed on the group-level, in which the models were fit to all 4608 trials (64 trials x 3 runs x 24 subjects) of the group’s collective data. Performing the model fit on the group data ensures consistent nodal function across the group and this approach is consistent with previous studies using HMM’s to estimate cognitive states from neuroimaging data [44].

Given that this procedure is sensitive to the initial parameter estimates, or “priors”, we solved each model using many different initial priors in order to more fully search the parameter space. After using 3000 different priors it became very unlikely to find a more optimal solution, so all models were solved using 3000 priors. After the Baum-Welch algorithm had been performed 3000 times with different priors, the solutions were then ranked by their final negative log-likelihood in order to find the most optimal solutions.

However, the unobserved nature of hidden nodes allows for symmetries to exist in the parameter space. Such symmetries occur because a node’s CPTs can be flipped, resulting in an equivalent solution as measured by the negative log-likelihood. In other words, the algorithm calculates a node be either 0 or 1, but we wish to impose the interpretation that 0 = “off/suppressed” and 1 = “on/enhanced”, rather than the alternative of 0 = “on/enhanced” and 1 = “off/suppressed”. Although symmetries aid in the finding of optimal solutions because they effectively shrink the parameter space, they become problematic when one wishes to interpret the solution. Therefore, we restricted the possible priors used to initialize the Baum-Welch algorithm in order to ensure the 0 = off/1 = on interpretation. For example, in the Cohen et al. model the detection of the scene being either neutral or threat-related should be positively correlated with whether the scene was actually neutral or threat-related. According to the negative log-likelihood, a solution in which these two are negatively correlated explains the data equally as well, but the solution in which these two are anticorrelated is nonsensical. Therefore, we only initialized using priors that are within the interpretable portion of the parameter space to ensure that a hidden state being either 0/1 confers the off/on interpretation. We also verified after the Baum-Welch procedure that the solution still represented the off/on interpretation and that the solution did not produce a trivial solution with highly correlated (r > 0.80) hidden nodes. These constraints were applied to each model and, therefore, do not alter the model comparisons, however, the constraints bias the final parameter values limiting inferences regarding particular parameters. For example, the target detector in the Mathews models is constrained to be positively correlated with the target itself, in order to insure the 0/1, off/on interpretation. Therefore, these parameters are biased by the constraints and should be subject to statistical tests of their reliability. However, the parameters governing responses are left unconstrained so stronger inferences regarding the quality of the model can be drawn when examining these parameters.

### 2.7 Model comparison and examination

Given that a more complex model should always provide a superior fit, and that the models under comparison varied in their complexity, we used four criteria to assess model fit: the Akaike Information Criterion (AIC) [53], AIC corrected for finite sample size (AICc) [54], Hannan-Quinn Information Criterion (HQC) [55], and the Bayesian Information Criterion (BIC) [56]. These criteria balance the achieved negative log-likelihood of a model with the number of parameters needed by that model. The criteria differ in how much they penalize additional parameters; the AIC applies the smallest penalty and the BIC applies the largest. Because we are selecting only the most optimal fit for each model, there is no variance with which to test the statistical significance of the superiority of one model over the other. Rather, we are simply comparing the models relatively using these parameters. However, by using multiple fit criteria we can obtain a degree of certainty regarding the relative ranking of the models. Additionally, the AICc can also be used to quantify how much better one model is than another in terms of how likely the worse model is actually better i.e. the relative likelihood. It is calculated as

$$Relative\;likelihood=exp(\frac{{AICc}_{min}-{AICc}_{j}}{2})$$ (1)

where AICc_min_ is the AICc of the optimal model and AICc_*i*_ is AICc of the next best model. This quantity expressed the likelihood that the next best model actually explains the data as well as the best model. Again, this value does not provide a statistical comparison between the model fits but provides a quantitative degree of certainty.

Additionally, we performed tests of the interpretability of the final model by testing for positive correlations between the actual target valence and the detected target valence and also the actual distractor valence and the detected distractor valence. To do so, for each participant separately we tested for correlation between these nodes and then used a simple one-tailed t-test across the group to test whether these correlations were significantly greater than 0. Having established this basic level of interpretability we referred directly to the conditional probability tables for further examination of the model.

### 2.8 Neural correlates of hidden nodes

Having applied the Baum-Welch algorithm to calculate the optimal parameters explaining the group’s data, we then applied the Viterbi algorithm to each individual’s set of data to obtain subject specific timecourses of the hidden states. The Viterbi algorithm uses the results of the Baum-Welch algorithm—the optimal model parameters (i.e. the arrows connecting the model nodes)—and the observed evidence (the stimulus features and reaction times) to explicitly solve for the most likely configuration of the hidden states for each trial (i.e. the nodes themselves) and the observed evidence (the stimulus features and the responses) [57]. In other words, the Viterbi algorithm calculates which nodes are inhibited and which are enhanced for each trial, providing a timecourse of each node’s activity. These timecourses were used as predictors of BOLD signal in a whole-brain voxelwise amplitude-modulated deconvolution implemented in AFNI (3dREMLfit). In addition to including the timecourses of the hidden states, the time course of the task stimuli and the reaction times themselves were also included to account for variance in brain activity related to the task itself and to improve the specificity of inferences regarding hidden state-related brain activity. The amplitude-modulated deconvolution tests whether brain activity of a given voxel is correlated with changes in the amplitude of the predictor. Here, for each trial the amplitude is determined by whether the node is off /suppressed (equal to 0) to on/enhanced (equal to 1). Thus, we are testing whether a given voxel encodes a particular hidden state. Following the deconvolution we used a robust one-tailed t test to test for consistent relationships between hidden state function and neural activity across the group. We then thresholded results at an uncorrected p < 0.005 and applied a clustering threshold (k = 23), based on the smoothness of the data using AFNI’s 3dClustSim, to obtain results significant at a corrected p < 0.05.

## Results

### 3.1 Task validation

Contrast-based task performance measures demonstrated the task to be a valid measure of implicit emotion regulation. Conflict, when considering all conflict conditions, regardless of whether threat was the target or distractor, did not significantly slow response times, although a trend was present for conflict related slowing (M = 5.0 ms; SE = 13.88; t = 2.04; p = 0.053). However, examining more specific contrasts reveals that this interference effect was driven by trials in which the distractor stimulus was threatening vs neutral (M = 65 ms; SE = 14.29; t = 4.23 p = 0.0003). Whereas conflict trials in which the threatening stimulus was the target were actually related to faster reaction times (M = -31 ms; SE = 14.29; t = -2.20; p = 0.038). Comparing conflict trials preceded by a nonconflict trial to those preceded by a conflict trial (i.e. Gratton effect) revealed significant adaptation to conflict (M = 15 ms; SE = 13.47; t = 2.07; p = 0.0499). This adaptation effect was strongest following trials in which the distractor was threat-related (M = 30 ms; SE = 6.53; t = 4.34; p = 0.0002), and was not present following trials in which the target was threat-related (M = -44 ms; SE = 21.64; t = -1.48; p = 0.152). We also tested for a slow emotional interference effect by examining whether threat-related stimuli altered responses on subsequent nonconflict neutral/neutral trials. Responses to neutral/neutral trials that were preceded by nonconflict threat/threat stimuli were significantly faster than those preceded by neutral/neutral stimuli (M = -38 ms; SE = 16.53; t = -3.45; p = 0.002). Tabulated results can be found in S1 Table. The previous analyses were performed having removed the incorrect trials. We performed additional analyses removing the incorrect trials and also trials occurring after an incorrect trial and found only very minimal changes to the results (S2 Table). These results support the validity of this novel task to probe the psychological effects of interest: emotional conflict, emotional conflict adaptation, and a slow emotional interference effect.

### 3.2 Model comparison

**Fig 2:**
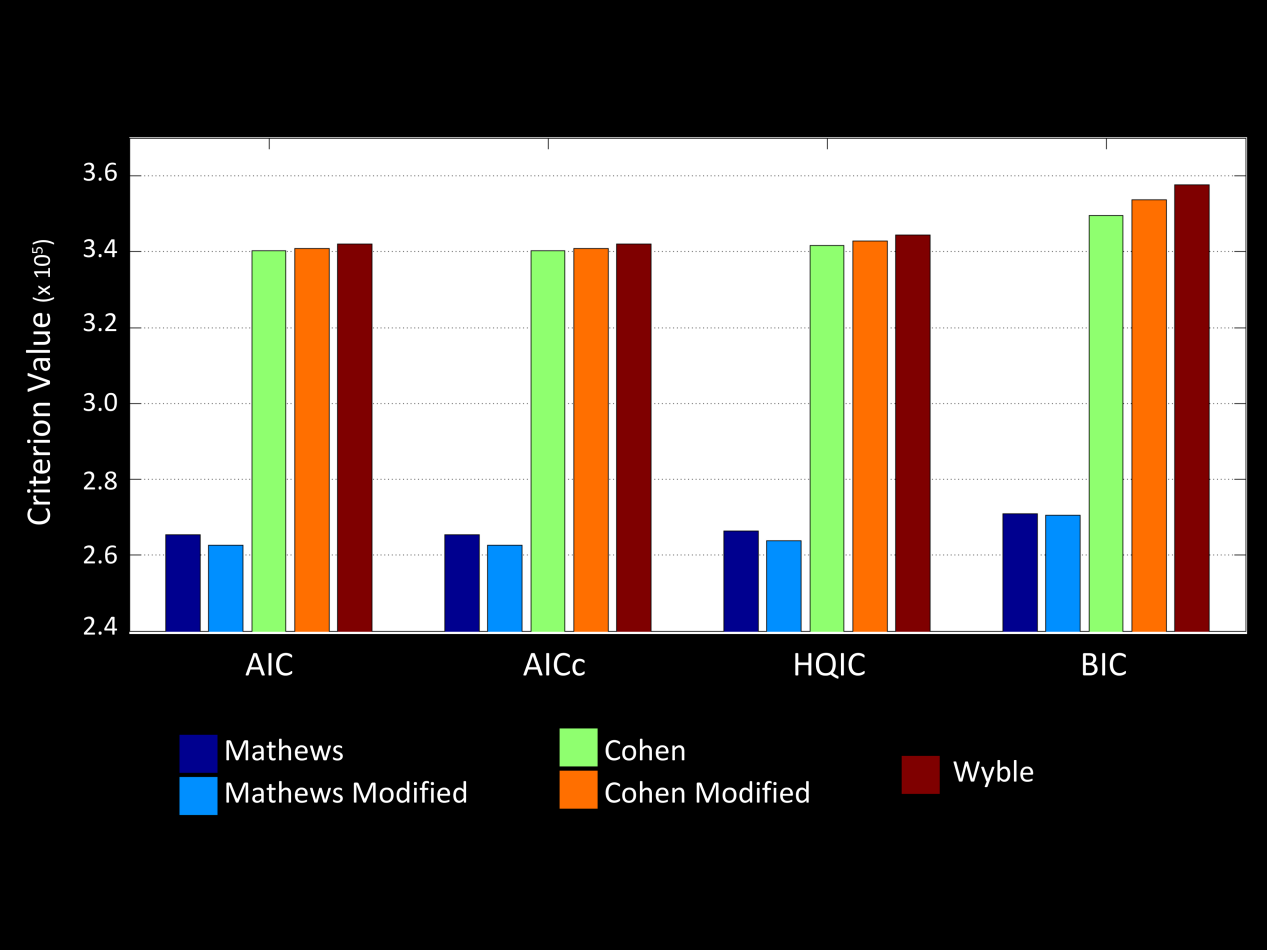
Model fit comparisons. Lesser values indicate better model fit. According to all criteria the modified Mathews et al. (1998) model fit best to the behavioral data. These values do not provide a statistical comparison between the model fits and has no variance, but they do provide a relative likelihood that one model is not actually better than the next. According to this value, the likelihood that the modified Mathews model is not actually best is 1.8 x 10^−64^.

According to all four fit criteria, the relative ranking of how well the data fit each model from best to worst was: Modified Mathews model > Mathews model > Cohen model > Modified Cohen model > Wyble model (Fig 2). Even according to BIC, which penalizes complexity the most, the data fit best to the modified Mathews model. Using the relative likelihood to compare the fit of the modified Mathews model to the fit of the next best model, the Mathews model, revealed that the relative likelihood that the Mathews model actually explains the data as well as the modified Mathews model (analogous to a *p* value) was 1.8 x 10^−64^, which is extremely significant and driven by the large number of observations (192 trials x 24 participants = 4608 observations).

When examining the interpretability of the resulting modified Mathews model we found that the target valence and target detector were positively correlated across the group (t = 32; p < 0.0001) and the distractor valence and distractor detector valence were also positively correlated across the group (t = 2.50; p = 0.01), suggesting interpretability of the model.

When examining the conditional probability tables of the model (S3 Table) we found also that task effort was more likely to be high following conflicting stimuli (0.68 for threatening target with neutral distractor and 0.74 for neutral target with threatening distractor) and high task effort biased the target detector to match the target (when task effort low 0.70 chance to detect threat when target actually neutral but 0.60 chance when task effort high; and 0.83 chance to accurately detector neutral regardless of task effort). Threat evaluation was likely to be high following dual threatening stimuli (0.94), and caused the distractor detector to be more likely to detect threat (when threat evaluation was high there was a 0.725 chance to accurately detector threatening distractor, when threat evaluation was low 0.463 chance to accurately detector threatening distractor). Also, when participants detected both stimuli to be neutral or the target to be threatening and the distractor neutral they were most likely to make a fast response (0.90 and 0.73, respectively), and were most likely to make a slow response when detecting a neutral target stimulus and a threatening distractor stimulus (0.83). These findings mirror the contrast-based analyses and suggest validity of the model to accurately capture implicit emotion regulation processes.

Additionally, to assess the reliability of this solution we tested whether the negative log-likelihoods of the other 2999 solutions were related to their difference from the optimal solution (as measured by the sum of the squared difference between each parameter and the optimal parameter from the best chosen solution). There was a significant negative relationship (beta = -0.004; p = 0.002) between negative log-likelihoods and distance from the optimal model indicating large scale stability of the solutions, such that solutions which fit the data better, according to the negative log-likelihood, were more similar to the optimal solution than solutions which fit the data worse.

### 3.3 Neural correlates of the modified Mathews model

The following is a description of the neural regions associated with each node of the model. A multiple regression was used in the deconvolution of the data, so that each effect listed below is significant when controlling for the other nodes in the model (e.g. the neural correlates of the Target detector are significant controlling for every other node, including the actual Target stimulus). Regions of interests are described in the following text and a full elaboration of significant clusters is provided in Table 1.

**Table 1:** Full description of significant clusters. All clusters reaching statistical significance at a whole brain corrected *p* < 0.05 (*t* > = 2.807, cluster threshold 23, k = 23). Coordinates are based on the MNI templates.

Reaction time	Region	Peak t- value	Size	Center of Mass		
				x	y	z
	dACC/pre-SMA	5.87	597	0	15	49
	R superior temporal sulcus	-5.74	510	51	-19	19
	L superior temporal sulcus	-5.36	501	-56	-16	17
	R anterior insula	5.34	343	40	17	7
	Midbrain	5.75	250	-4	-19	-5
	L anterior insula	5.54	208	-37	18	7
	R Precentral gyrus	-6.08	156	17	-36	66
	L posterior MFG	5.31	131	-43	10	38
	R Cuneus	5.42	94	3	-79	6
	R caudate	4.57	89	10	-3	13
	L supramarginal gyrus	4.43	88	-42	-49	48
	L postcentral gyrus	-5.30	86	-22	-41	65
	L posterior SFG	4.81	82	-30	-9	57
	R posterior MFG	4.00	55	45	9	35
	R posterior MFG	4.56	28	36	23	32
	L anterior MFG	4.29	26	-31	48	24
	L supramarginal gyrus	4.15	25	-22	-70	47
Target detector	Region	Peak t- value	Size	Center of Mass		
				x	y	z
	R anterior insula	8.65	108	47	14	9
	L precentral gyrus	-6.20	108	-40	-27	58
	L anterior insula	3.97	70	-37	16	6
	Midbrain	6.99	68	-1	-22	-8
	Posterior cingulate cortex	-3.64	27	-6	-52	26
Distractor detector	Region	Peak t- value	Size	Center of Mass		
				x	y	Z
	R inferior cuneus	-4.18	26	26	-82	-16
Task effort	Region	Peak t- value	Size	Center of Mass		
				X	y	z
	R anterior insula	-6.17	153	39	16	7
	dACC	-6.54	89	-4	15	33
	L anterior insula	-6.04	85	-34	17	9
	Thalamus	-5.57	49	5	-19	1
	Midbrain	-4.14	25	2	-26	-17
Threat evaluation	Region	Peak t- value	Size	Center of Mass		
				x	y	z
	R supramarginal gyrus	5.81	108	42	-53	43
	R anterior insula / white matter	5.56	26	40	19	18
Target stimulus	Region	Peak t-value	Size	Center of Mass		
				X	y	z
	L lingual gyrus	-7.02	828	-8	-73	-1
	R ventral visual cortex	11.00	804	42	-67	-4
	L ventral visual cortex	8.98	574	43	-63	-6
	R parahippocampus	-5.21	53	22	-41	-6
	R lingual gyrus	-5.40	46	13	-53	10
	L anterior insula / white matter	4.45	25	-27	13	4
	L supramarginal gyrus	-5.01	24	-17	-67	42
	R parietooccipital sulcus	4.39	24	23	-76	38
Distractor stimulus	Region	Peak t-value	Size	Center of Mass		
				x	y	z
	R ventral visual cortex	9.56	1020	39	-67	-3
	L ventral visual cortex	8.52	870	-42	-66	-3
	Medial visual cortex / lingual gyri	-6.28	660	-2	-72	3
	R lateral SMA	-10.50	135	53	-7	31
	L posterior IFG	-5.28	87	-58	1	12
	R putamen	-5.81	59	27	10	6
	R precentral gyrus	-5.65	53	39	-22	58
	L posterior MFG	4.56	47	-39	8	34
	R posterior insula	-5.39	43	48	-29	15
	L supramarginal gyrus	5.37	42	-28	-69	52
	R middle insula	-4.01	38	39	-7	12
	L posterior insula	-4.69	35	-30	-15	6
Event occurred	Region	Peak t- value	Size	Center of Mass		
				X	y	z
	Broad positive and negative areas	n/a	16582	-5	-30	21
	R anterior insula	7.89	568	40	15	22
	R supramarginal gyus	-7.61	365	49	-61	35
	L anterior insula	9.58	286	-38	12	11
	L anterior IFG	-5.49	232	-49	33	8
	R anterior IFG	-5.76	93	47	35	3
	L posterior insula	4.61	72	-49	-22	22
	L putamen	5.73	65	-25	4	4
	R pre-SMA	4.49	46	33	-6	60
	R hippocampus	-4.85	37	23	-17	-18
	Corpus callosum (white matter)	5.14	30	-8	-28	27

#### 3.3.1 Reaction time

**Fig 3:**
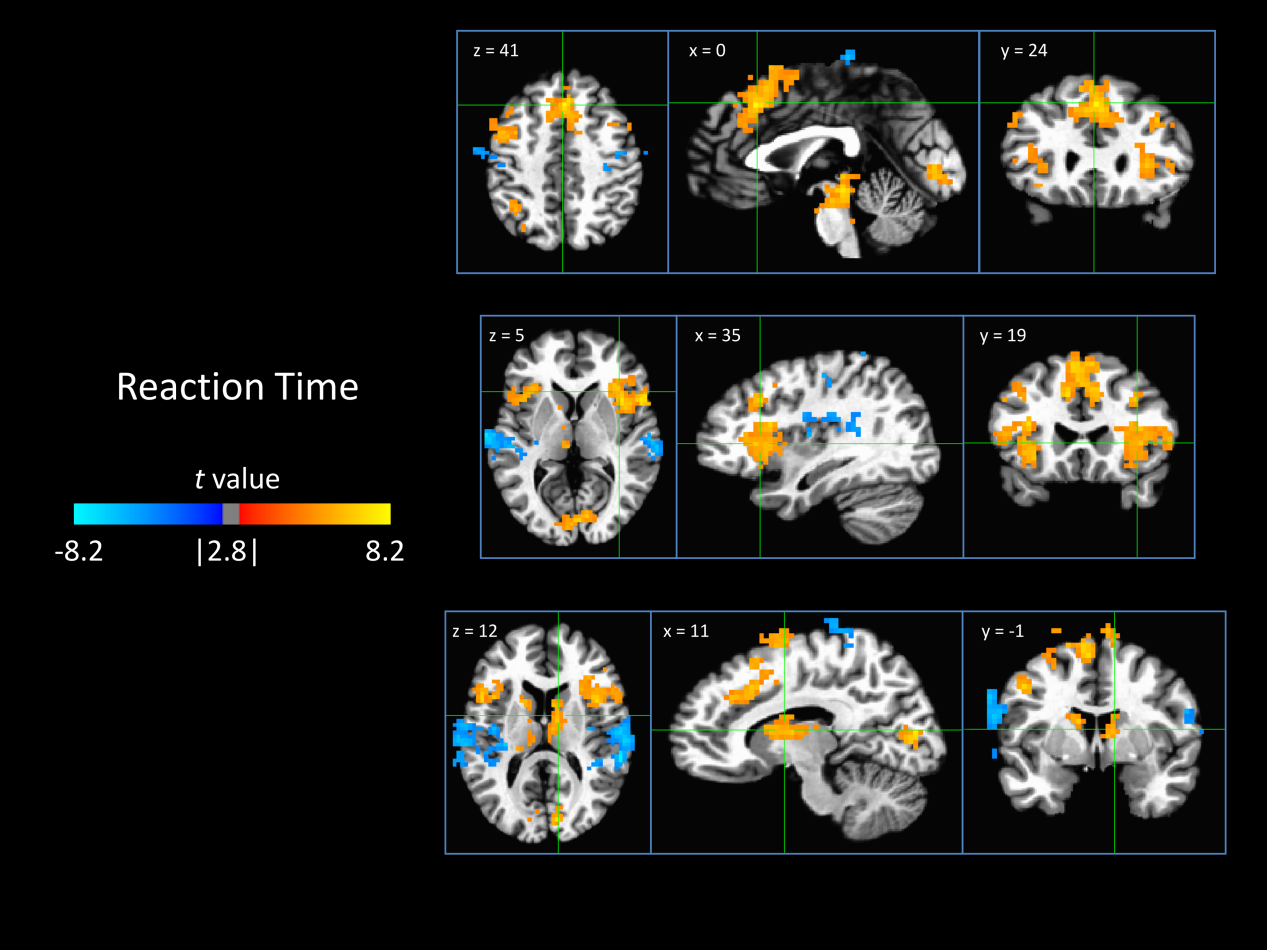
Neural encoding of reaction time. Results are from a multiple regression amplitude-modulated deconvolution and depict areas where activity scaled significantly with reaction time. Positive values (orange) indicate that greater activity was associated with longer reaction times. Likewise, negative values (blue) are areas where activity was inversely related to reaction time.

Longer reaction time was positively related to broad areas of activity in the bilateral anterior insula cortex, the dACC/pre-SMA, and bilateral caudate. It was negatively related to activity in bilateral posterior insula (Fig 3).

#### 3.3.2 Target detector

**Fig 4:**
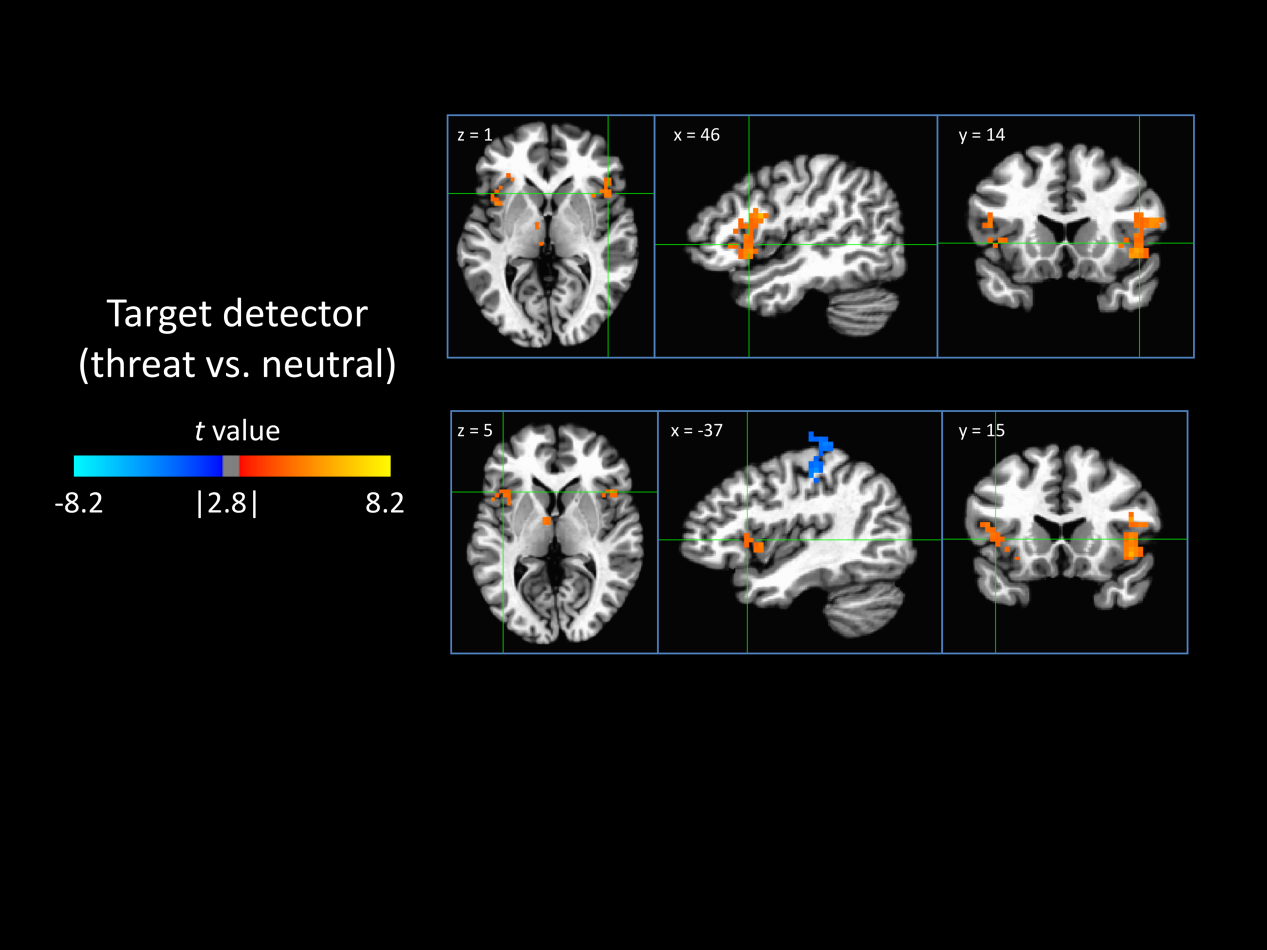
Neural encoding of the detection of the target stimulus. Shown are areas that were differentially responsive to the detection of threatening versus neutral target stimuli, indicating a neural correlate of the distractor detector. Positive values (orange) indicate that a region was activated in response to the detection of a threatening target and/or deactivated by the detection of a neutral target. Likewise, the negative values (blue) follow the inverse of this relationship.

The model-based detection of a threat-related target stimulus was associated with increased activity in the bilateral anterior insula and decreased activity in the left motor cortex (Fig 4).

#### 3.3.3 Distractor detector

**Fig 5:**
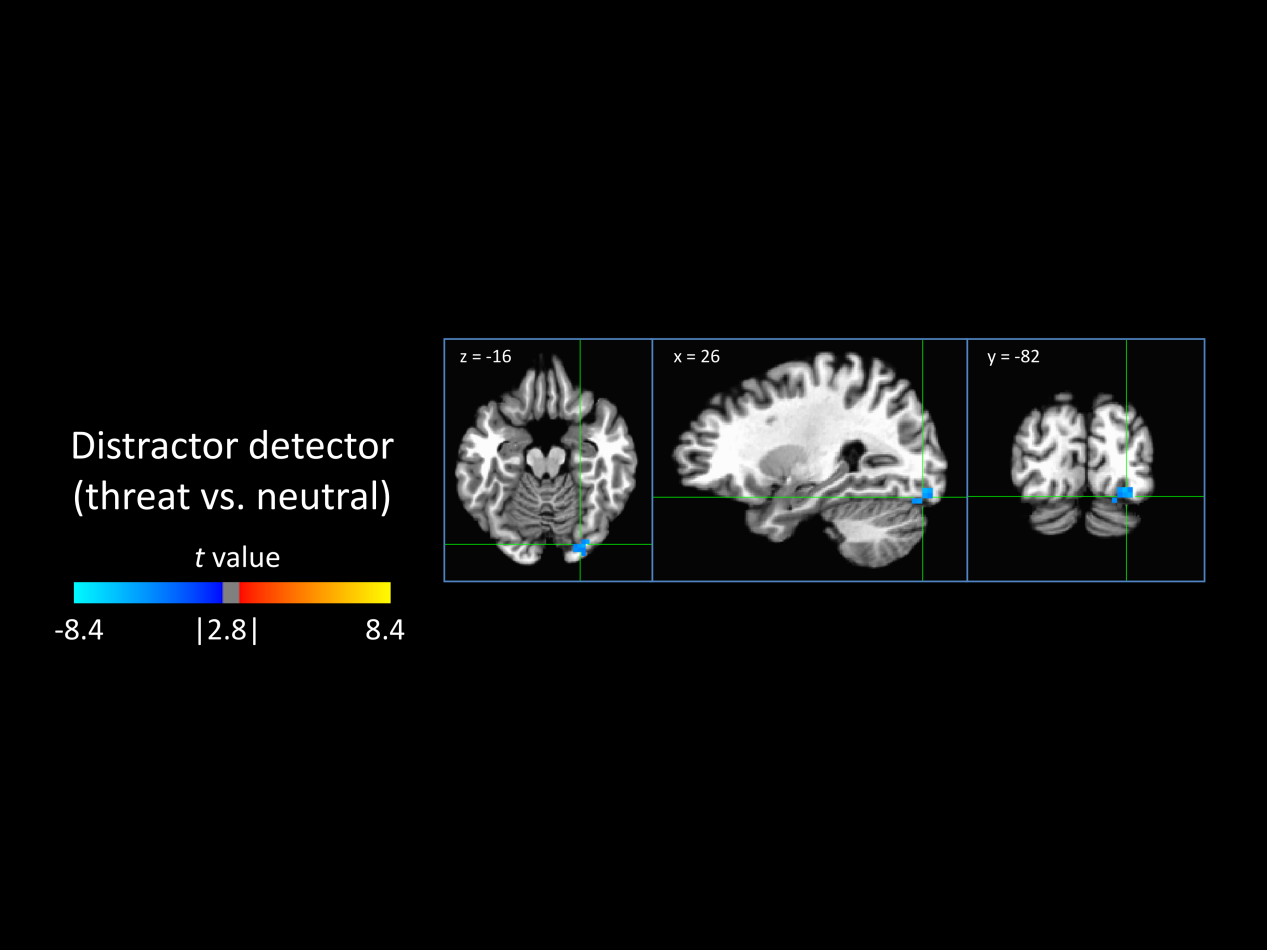
Neural encoding of the detection of the distractor stimulus. Shown is an area that was differentially responsive to the detection of threatening versus neutral distractor stimuli, indicating a neural correlate of the distractor detector. The negative values (blue) indicate that this region was deactivated in response to the detection of a threatening target and/or activated by the detection of a neutral target.

The model-based detection of a threat-related stimulus in the distractor was not related to activity in any nodes in the salience network but only a small cluster of decreased activity in the inferior visual cortex (Fig 5).

#### 3.3.4 Task effort

**Fig 6:**
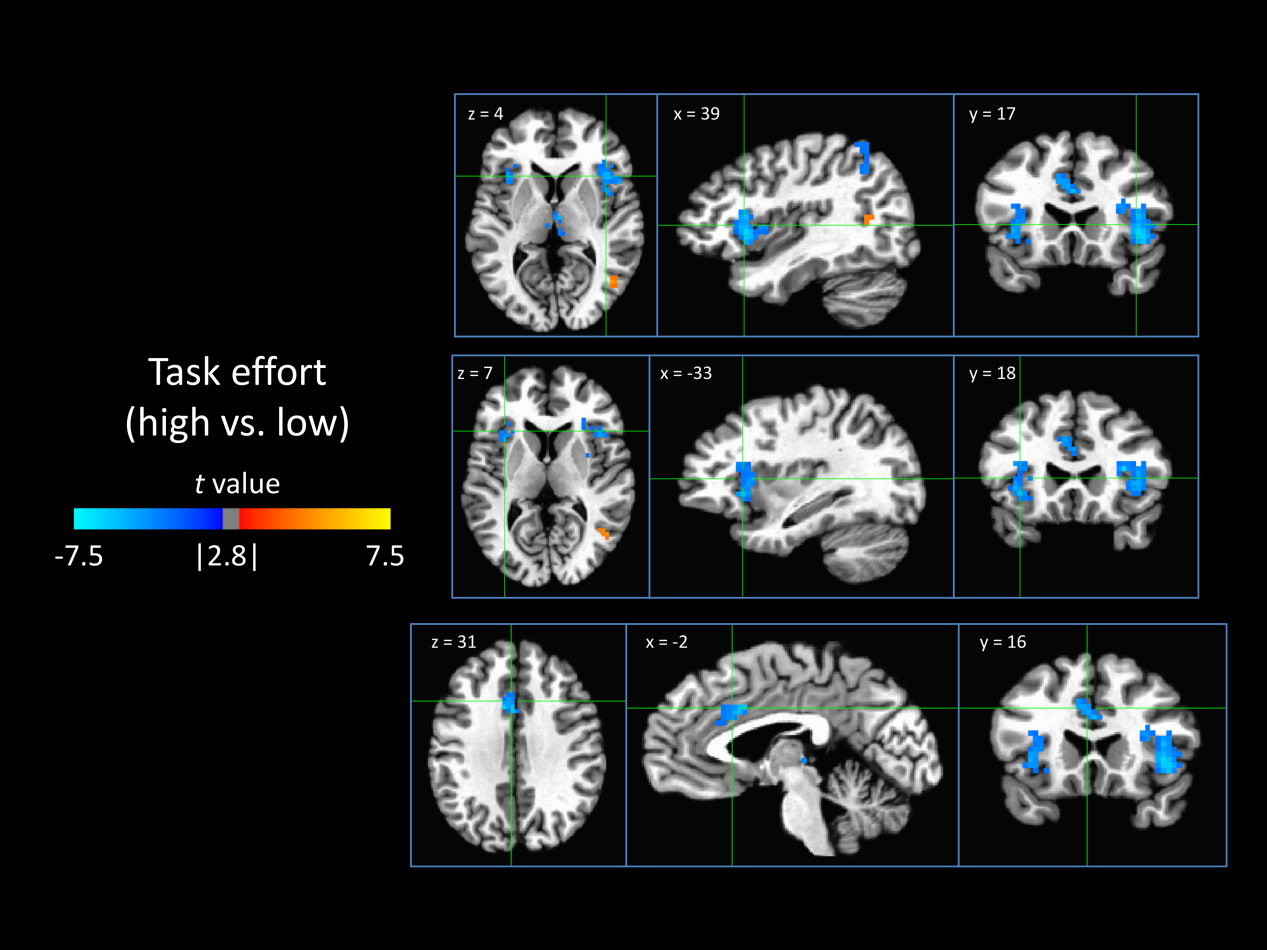
Neural encoding of task effort. Shown are areas that were differentially responsive to high task effort versus low task effort, indicating a neural correlate of the task effort node. Negative values (blue) indicate that a region was deactivated in response to high task effort and/or activated by low task effort.

Model-based high task effort (high compared to low) was associated with decreases in the nodes comprises the salience network—the dACC and the bilateral anterior insula (Fig 6).

#### 3.3.5 Threat evaluation

**Fig 7:**
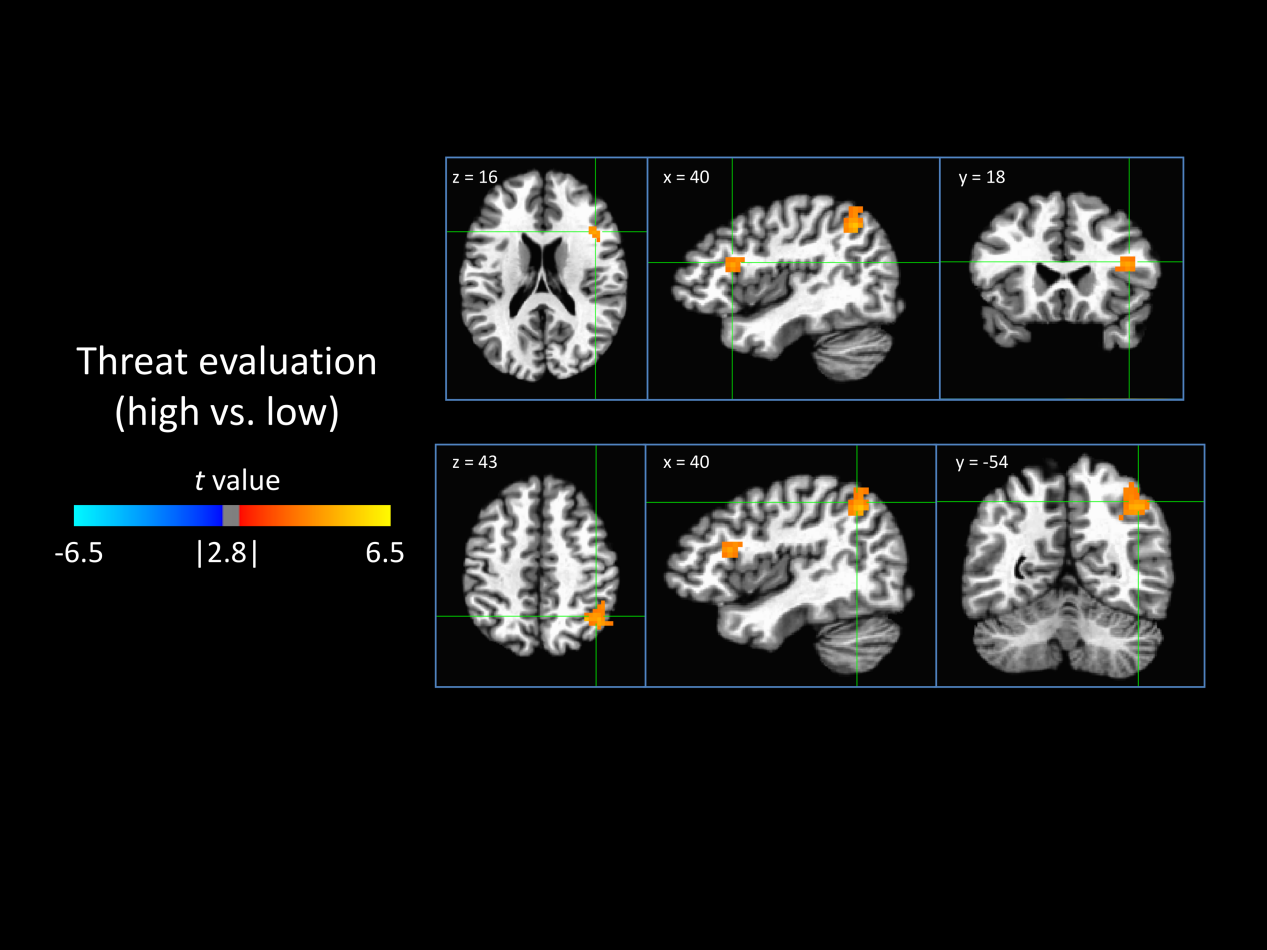
Neural encoding of threat evaluation. Shown are areas that were differentially responsive to high threat evaluation versus low threat evaluation, indicating a neural correlate of the threat evaluation node. Positive values (orange) indicate that a region was activated in response to high threat evaluation and/or deactivated by low threat evaluation.

Model-based threat evaluation (high compared to low) was associated with greater activity in the right anterior insula and the right parietal cortex (Fig 7).

#### 3.3.6 Target stimulus

Threatening target stimuli (compared to neutral target stimuli) evoked exaggerated processing in the ventral visual cortex and decreases in the medial visual cortex extending into the parahippocampus. Additionally, threatening targets were associated with increased activity in a small cluster in the left anterior insula (S1 Fig).

#### 3.3.7 Distractor stimulus

The presence of threat in the distractor stimulus (compared to neutral) was associated with a similar pattern of activation in the ventral visual and medial cortices, but also associated with deactivation in the posterior and insula (S2 Fig).

#### 3.3.8 Event occurred

The baseline intercept of the regression accounts for neural activity related to the occurrence of all trials, regardless of type. This baseline of activity contained both positive and negative areas of activation broadly throughout the entire brain.

## Discussion

The study of automatic biases in cognitive and emotional processing has a long history of using models to formulate and test hypotheses [19, 21, 29, 30]. However, these models have only been indirectly tested through the use of simulations, which test for modeled behavior that corresponds with human behavior. Here we sought to use Hidden Markov Modeling to test previously hypothesized models of implicit emotion regulation on actual human data by first comparing the models and then testing for neural correlates of the model which provided the best explanation of the data.

According to all four goodness-of-fit criteria, the model that best fit the data was the modified Mathews model, and both the modified and the unmodified Mathews models greatly outperformed the other models. The Mathews models differ from the other models in two respects. They do not require knowledge as to which stimulus feature is the target and which is the distractor, and are therefore able to specify an attention-biasing system that is specific to the distractor stimuli, referred to as the threat evaluation system.

The additional complexity of the Cohen model and Wyble model used to specify which feature is the target and which is the distractor is likely not necessary in this task; the valence of the target and distractor is sufficient to capture the relevant features of the stimuli. However, this framework is likely only applicable to tasks in which both features of the stimuli cause a similar interference effect. For example, in the classic Stroop task participants demonstrate an interference effect when naming the ink color, but there is no interference when reading the word, which is presumably due to the fact that word-read has higher automaticity than color naming [58]. Applying the Mathews models to the classic Stroop task, therefore, would likely result in a poor fit because they cannot account for difference in the processing of the two stimulus features. The Cohen models would likely produce a better fit to data generated using such a task. In this respect, the Cohen models are possibly more generalizable, because they can accommodate tasks in which processing of the stimulus features occurs with different automaticity, but on tasks in which automaticity is comparable, such as the task used here, this additional complexity is not needed. However, the threat evaluation system of the Mathews models does appear to add useful complexity to the models. This threat evaluation system controls attentional bias toward threatening distractor stimuli. The high performance of the models which included this node suggests that the allocation of attention toward relevant emotional stimuli is independent from the allocation of attention away from distractor stimuli.

The modification to the Mathews et al. model provided even greater performance in explaining the behavioral data. This modification adds a temporal effect to the model, allowing detectors of the current trial to inform the task effort and threat evaluation system of the subsequent trial. Given the well-established observations of conflict adaptation [24, 25] and slow interference effects [26–28], which was also observed in our task validation, it is not surprising that this modification led to better model performance. However, this modification did not improve the fit of the Cohen model. Combined with the poor indices of fit, this further suggests that the Cohen model provides a poor fit to the observed data from an emotional conflict task. The Wyble model performed the worst of the five models, which was likely due to the complexity of the model. This complexity allows the model to accommodate any possible conflict task and accounts for temporal effects, and while this high degree of complexity does not hinder the model in simulations, it becomes untenable when all aspects of the complex models are estimated solely from reaction time data. One potential method to improve the fit of the models, especially the more complex models, is to use other indirect measures of the hidden nodes, such as neural activity or skin conductance responses as trial-by-trial indicators of a particular node’s activity. For example, skin conductance measures from each trial could be used to obtain more accurate estimates of the negative emotional state in the Wyble model, or dlPFC neural activity could be used as an indicator of the task effort node in the Mathews model.

We also observed large scale stability in the fit of the modified Mathews to the data. The optimal parameters were chosen by ranking the solutions according to their negative log-likelihoods and finding the best solution in which the hidden states were not highly correlated and the detectors correlated positively with their stimuli, which ensured the 0/1, off/on, interpretation. Having determined the optimal solution, we found that the negative log-likelihoods of the remaining solutions were correlated with the degree to which the solutions differed from the optimal solution. This suggests that there is high-level structure in the solutions of the model and that with increasing model fit, the solutions converge toward what we have found to be the optimal solution. The parameters of this model also confirm and account for previously observed implicit emotion regulation processes, further indicating the validity of the found solution. Although most of these parameters are biased by the initialization constraints used to insure interpretability of the model, the response parameters were not constrained but they still converged to parameters which are concordant with the contrast based analyses, such that the largest impairments in reaction times were due to stimuli in which the target was neutral and the distractor was threat-related. This provides further evidence of the ability of the model to accurately capture implicit emotion regulation processes.

The test for neural correlates of the optimal solution to the modified Mathews model revealed that activity in regions previously implicated in implicit emotion regulation were associated with the hidden nodes of the model. Importantly, these neural correlates of the hidden states are significant when controlling for variance attributable to reaction time as well as the actual valence of the stimuli. Therefore, these effects are not related simply to the presence of threat or the overt behavioral response to it, but to how that threat is processed according to the model.

Longer reaction time was associated with large areas of activity in the cingulate and insula, consistent with previous findings that activity in these areas scales positively with reaction time, suggesting their role in sustained attention and awareness [59, 60]. The actual presence of the threat in both the target and the distractor stimuli evoked greater activity in a large portion of the ventral visual cortex, which has been shown to be related to identification of visual stimuli (‘What’ visual stream) [61, 62]. This suggests that both threatening stimuli, regardless of their relevance to task demands, garner similar levels of processing towards determining the identity of a threatening stimulus. In addition, the threatening target stimuli themselves (i.e. not the model based detection of threat) were associated with a small cluster of increased activity in the left anterior insula, suggesting increased awareness to the threat stimuli at a further upstream stimulus processing level, irrespective of their effects on behavior.

When examining the neural correlates of the modified Mathews model we found that several nodes of the model were associated with activity in the anterior insula and dACC. In particular, the detection of threat, compared to neutral in the target stimulus was associated with increased activity in the anterior insula bilaterally, while high task effort, which improves the accuracy of the target detector, was associated in inhibition of activity in these same areas as well as inhibition of the dACC. Given also that activity in these areas was related to longer reaction times, this suggests that the threatening target stimuli represented salient environmental cues and that this salience distracted attention away from the task making the motor response identifying the valence. Additionally, high task effort, which increased the accuracy of the target detector, was associated with reduction in the activity of these areas, suggesting that identification of the valence of the targets was facilitated by reduction in the perceived salience of the images. That is, the participants may have abstracted the emotional content of the images in order to identify the valence without being distracted by it.

With regard to the distractor detector and threat evaluation nodes of the model, the detection of threatening distractor stimuli was not associated with greater activity in any brain region of interest. The task was counterbalanced with respect to which stimulus feature, the face or the scene, was the target so this is not explainable by imbalance in task design or feature valence. It may suggest that since the distracting stimuli were irrelevant they engendered less of an emotional response. The present analyses did not investigate neural changes associated with specific interactions between the target and distractor, e.g. detecting a threatening distractor when detecting the target as threatening vs. neutral, and future studies could analyze whether such interactions may better explain neural activity related to the distracting stimuli. We did find, however, that high threat evaluation was associated with greater activity in a small region in the right superior anterior insula. The location, size, and laterality of the finding preclude strong interpretation but may suggest that greater detection of threat is associated with a baseline higher arousal level.

Although previous research strongly implicates the amygdala as an important node in the emotion processing neurocircuitry, this region was not related to any of the hidden nodes of the emotion regulation model. This is most likely due to habituation effects in the amygdala, whereby amygdala responses diminish with repeated exposure to similar threat-related stimuli [63]. Therefore, the lack of amygdala activity is possibly due to the length of the task. We also expected to find evidence for a role of the DLPFC and pregenual ACC in the modified Mathews model, particularly with the ‘Task effort’ and ‘Threat evaluation’ subprocesses, respectively. However, we did not find evidence that either region was related to the model. This could indicate that the roles of these regions in this behavioral process are not compatible with the representation of this behavioral process by the modified Mathews model. This does not preclude these regions from being involved, but suggests that they are involved with some other, perhaps higher-order, behavioral processes.

Among this group of developing adolescents we have found evidence that the model of emotion regulation most supported by human data is the modified Mathews model. These findings confirm previous evidence that emotion regulation is a dynamic process of continual adaptation to relevant and irrelevant threatening signals. Additionally, they suggest that the direction of attention toward goal-relevant threat is independent of the direction of attention away from a goal-irrelevant threat, but that these two processes drawn upon a common neural system, the salience network, which acts to balance goal-directed behavior with automatic responses to biologically-relevant cues.

Being the first study to apply and test previously hypothesized models of implicit emotion regulation to actual human data using a Hidden Markov modeling framework, the work presented is not without limitations. The use of a sample of adolescent girls impairs generalizability to adults and further studies among healthy adult populations are needed to further validate the approach described here. However, a recent study of emotional conflict in adolescents age 10–15 found that age correlated with conflict-related activity in the middle frontal gyri, and not the regions found in the present study [64], suggesting that the regions found here are not affected by age. Also, this data was acquired as part of a larger study of adolescent assault exposure and the use of this sample facilitates future study of the neural and cognitive mechanisms affected by assault exposure and may ultimately contribute to more efficacious early interventions for adolescent assault victims, which are needed to lessen the burden of mental illness among assault victims. Additionally, the absence of a baseline nonemotional task precludes the ability to infer that the findings are strictly due to the emotional content of the stimuli and the specificity of these findings for emotional versus cognitive processes is a potential area of future research. The implementation using the HMM framework necessitated the discretization of the hidden nodes and the response values. More sophisticated techniques can model nodes continuously, but are more complex and require a greater number of parameters to describe the models. Assuming a linear relationship between nodal activity and its influence on its neighboring nodes, discretizing nodal activity only reduces the power to detect a relationship and does not affect the magnitude or direction of the relationship. An additional limitation is that, while we have modeled temporal dynamics between trials, we did not model within-trial temporal dynamics. Such within-trial temporal effects were used in the simulation studies testing the models’ ability to simulate human behavior. However, the approach used here is not conceptually incompatible with temporal within-trial effects and such temporal models could be constructed and tested, although this would necessitate a higher temporal resolution of observations than obtained here. A technique such as magnetoencepahlography (MEG) could be used to obtain high-temporal resolution measures of nodal activity and provide causal temporal structure within the response to a single trial (i.e. real-time nodal interactions). Additionally, this framework could potentially offer more elaborate explanations of developmental and mental-illness related changes in emotion regulation.

## Supporting information

S1 Fig Neural encoding of the target stimulus. Shown are areas that were differentially responsive to the actual presence of threatening versus neutral target stimuli (not the detection of the stimuli). Positive values (orange) indicate that a region was activated in response to threatening target stimuli and/or deactivated by neutral target stimuli. Likewise, the negative values (blue) follow the inverse of this relationship.
(TIF)

S2 Fig Neural encoding of the distractor stimulus. Shown are areas that were differentially responsive to the actual presence of threatening versus neutral distractor stimuli (not the detection of the stimuli). Positive values (orange) indicate that a region was activated in response to threatening target stimuli and/or deactivated by neutral target stimuli. Likewise, the negative values (blue) follow the inverse of this relationship.
(TIF)

S1 Table Contrast-based measures of normative task performance. (DOCX)

S2 Table Contrast-based measures of normative task performance–excluding incorrect trials and their subsequent trials. (DOCX)

S3 Table Optimal model parameters. (DOCX)

## References

[1] Frijda NH. The current status of emotion theory. Bulletin of the British Psychological Society. 1986;39:A75-A. PubMed PMID: WOS:A1986C479300082.

[2] John OP, Gross JJ. Healthy and unhealthy emotion regulation: Personality processes, individual differences, and life span development. J Pers. 2004;72(6):1301–33. doi: 10.1111/j.1467-6494.2004.00298.x PubMed PMID: WOS:000224756300008. 15509284 10.1111/j.1467-6494.2004.00298.x

[3] Gross JJ. Emotion regulation: Affective, cognitive, and social consequences. Psychophysiology. 2002;39(3):281–91. doi: 10.1017.S0048577201393198 PubMed PMID: WOS:000175419200002. 12212647 10.1017/s0048577201393198

[4] Egner T, Etkin A, Gale S, Hirsch J. Dissociable Neural Systems Resolve Conflict from Emotional versus Nonemotional Distracters. Cerebral Cortex. 2008;18(6):1475–84. doi: 10.1093/cercor/bhm179 17940084 10.1093/cercor/bhm179

[5] Etkin A, Egner T, Peraza DM, Kandel ER, Hirsch J. Resolving Emotional Conflict: A Role for the Rostral Anterior Cingulate Cortex in Modulating Activity in the Amygdala. Neuron. 2006;51(6):871–82. doi: 10.1016/j.neuron.2006.07.029 16982430 10.1016/j.neuron.2006.07.029

[6] Cisler JM, Wolitzky-Taylor KB, Adams TG Jr, Babson KA, Badour CL, Willems JL. The emotional Stroop task and posttraumatic stress disorder: A meta-analysis. Clinical Psychology Review. 2011;31(5):817–28. doi: 10.1016/j.cpr.2011.03.007 21545780 10.1016/j.cpr.2011.03.007 PMC3132173

[7] Etkin A, Schatzberg AF. Common Abnormalities and Disorder-Specific Compensation During Implicit Regulation of Emotional Processing in Generalized Anxiety and Major Depressive Disorders. American Journal of Psychiatry. 2011;168(9):968–78. doi: 10.1176/appi.ajp.2011.10091290 21632648 10.1176/appi.ajp.2011.10091290

[8] Zeman J, Cassano M, Perry-Parrish C, Stegall S. Emotion regulation in children and adolescents. J Dev Behav Pediatr. 2006;27(2):155–68. doi: 10.1097/00004703-200604000-00014 PubMed PMID: WOS:000237609900012. 16682883 10.1097/00004703-200604000-00014

[9] Somerville LH, Jones RM, Casey BJ. A time of change: Behavioral and neural correlates of adolescent sensitivity to appetitive and aversive environmental cues. Brain and Cognition. 2010;72(1):124–33. doi: 10.1016/j.bandc.2009.07.003 PubMed PMID: WOS:000274128400013. 19695759 10.1016/j.bandc.2009.07.003 PMC2814936

[10] Finkelhor D, Ormrod RK, Turner HA. Polyvictimization and trauma in a national longitudinal cohort. Development and Psychopathology. 2007;19(1):149–66. doi: 10.1017/S0954579407070083 PubMed PMID: WOS:000244114100008. 17241488 10.1017/S0954579407070083

[11] Finkelhor D, Turner H, Ormrod R, Hamby SL. Violence, Abuse, and Crime Exposure in a National Sample of Children and Youth. Pediatrics. 2009;124(5):1411–23. doi: 10.1542/peds.2009-0467 19805459 10.1542/peds.2009-0467

[12] Nooner KB, Linares LO, Batinjane J, Kramer RA, Silva R, Cloitre M. Factors Related to Posttraumatic Stress Disorder in Adolescence. Trauma Violence & Abuse. 2012;13(3):153–66. doi: 10.1177/1524838012447698 PubMed PMID: WOS:000305533600002. 22665437 10.1177/1524838012447698

[13] Kilpatrick DG, Acierno R, Saunders B, Resnick HS, Best CL, Schnurr PP. Risk factors for adolescent substance abuse and dependence: Data from a national sample. Journal of Consulting and Clinical Psychology. 2000;68(1):19–30. doi: 10.1037//0022-006x.68.1.19 PubMed PMID: WOS:000085495600004. 10710837 10.1037//0022-006x.68.1.19

[14] Kilpatrick DG, Ruggiero KJ, Acierno R, Saunders BE, Resnick HS, Best CL. Violence and risk of PTSD, major depression, substance abuse/dependence, and comorbidity: Results from the National Survey of Adolescents. Journal of Consulting and Clinical Psychology. 2003;71(4):692–700. doi: 10.1037/0022-006x.71.4.692 12924674 10.1037/0022-006x.71.4.692

[15] Cisler JM, Steele JS, Smitherman S, Lenow JK, Kilts CD. Neural processing correlates of assaultive violence exposure and PTSD symptoms during implicit threat processing: A network-level analysis among adolescent girls. Psychiatry Research-Neuroimaging. 2013;214(3):238–46. doi: 10.1016/j.pscychresns.2013.06.003 PubMed PMID: WOS:000327531600009. 23969000 10.1016/j.pscychresns.2013.06.003 PMC3852193

[16] Weinberg A, Klonsky ED. Measurement of emotion dysregulation in adolescents. Psychological Assessment. 2009;21(4):616–21. doi: 10.1037/a0016669 19947794 10.1037/a0016669

[17] Dyer F. The Stroop phenomenon and its use in the stlldy of perceptual, cognitive, and response processes. Memory & Cognition. 1973;1(2):106–20. doi: 10.3758/bf03198078 24214501 10.3758/BF03198078

[18] Stroop JR. Studies of interference in serial verbal reactions. Journal of Experimental Psychology. 1935;18(6).

[19] Cohen JD, Dunbar K, McClelland JL. On the control of automatic processes—A parallel distributed-processing account of the Stroop effect. Psychological Review. 1990;97(3):332–61. doi: 10.1037//0033-295x.97.3.332 PubMed PMID: WOS:A1990DN33800002. 2200075 10.1037/0033-295x.97.3.332

[20] Macleod CM. Half a century of research on the Stroop effect—An integrative review. Psychological Bulletin. 1991;109(2):163–203. doi: 10.1037//0033-2909.109.2.163 PubMed PMID: WOS:A1991FA63300001. 2034749 10.1037/0033-2909.109.2.163

[21] Mathews A, Mackintosh B. A cognitive model of selective processing in anxiety. Cognitive Therapy and Research. 1998;22(6):539–60. doi: 10.1023/a:1018738019346 PubMed PMID: WOS:000077736100002.

[22] Mathews A, Macleod C. Cognitive approaches to emotion and emotional disorders. Annual Review of Psychology. 1994;45:25–50. doi: 10.1146/annurev.ps.45.020194.000325 PubMed PMID: WOS:A1994MV48600003. 8135504 10.1146/annurev.ps.45.020194.000325

[23] Williams JMG, Mathews A, MacLeod C. The emotional stroop task and psychopathology. Psychological Bulletin. 1996;120(1):3–24. doi: 10.1037/0033-2909.120.1.3 PubMed PMID: WOS:A1996UT74800001. 8711015 10.1037/0033-2909.120.1.3

[24] Gratton G, Coles MGH, Donchin E. Optimizing the use of information—Strategic control of activation of responses. Journal of Experimental Psychology-General. 1992;121(4):480–506. doi: 10.1037//0096-3445.121.4.480 PubMed PMID: WOS:A1992JY18600010. 1431740 10.1037//0096-3445.121.4.480

[25] Sheth SA, Mian MK, Patel SR, Asaad WF, Williams ZM, Dougherty DD, et al Human dorsal anterior cingulate cortex neurons mediate ongoing behavioural adaptation. Nature. 2012;488(7410):218-+. doi: 10.1038/nature11239 PubMed PMID: WOS:000307267000035. 22722841 10.1038/nature11239 PMC3416924

[26] Tzelgov J, Henik A, Berger J. Controlling Stroop effects by manipulating expectations for color words. Memory & Cognition. 1992;20(6):727–35. doi: 10.3758/bf03202722 PubMed PMID: WOS:A1992JX88100013. 1435275 10.3758/bf03202722

[27] McKenna FP, Sharma D. Reversing the emotional stroop effect reveals that it is not what it seems: The role of fast and slow components. Journal of Experimental Psychology-Learning Memory and Cognition. 2004;30(2):382–92. doi: 10.1037/00278-7393.30.2.382 PubMed PMID: WOS:000189184500008. 10.1037/0278-7393.30.2.382 14979812

[28] Phaf RH, Kan K-J. The automaticity of emotional Stroop: A meta-analysis. J Behav Ther Exp Psychiatry. 2007;38(2):184–99. doi: 10.1016/j.jbtep.2006.10.008 PubMed PMID: WOS:000246319500008. 17112461 10.1016/j.jbtep.2006.10.008

[29] Botvinick MM, Braver TS, Barch DM, Carter CS, Cohen JD. Conflict monitoring and cognitive control. Psychological Review. 2001;108(3):624–52. doi: 10.1037//0033-295x.108.3.624 PubMed PMID: WOS:000170892100006. 11488380 10.1037/0033-295x.108.3.624

[30] Wyble B, Sharma D, Bowman H. Strategic regulation of cognitive control by emotional salience: A neural network model. Cognition Emotion. 2008;22(6):1019–51. doi: 10.1080/02699930701597627 PubMed PMID: WOS:000257752700002.

[31] Carter CS, Macdonald AMI, Stenger VA, Cohen JD. Dissociating the contributions of DLPFC and anterior cingulate to executive control: An event-related fMRI study. Brain and Cognition. 2001;47(1–2):66–9. PubMed PMID: WOS:000172021100055.

[32] Egner T, Hirsch J. The neural correlates and functional integration of cognitive control in a Stroop task. NeuroImage. 2005;24(2):539–47. doi: 10.1016/j.neuroimage.2004.09.007 15627596 10.1016/j.neuroimage.2004.09.007

[33] Kerns JG, Cohen JD, MacDonald AW, Cho RY, Stenger VA, Carter CS. Anterior Cingulate conflict monitoring and adjustments in control. Science. 2004;303(5660):1023–6. doi: 10.1126/science.1089910 PubMed PMID: WOS:000188918000049. 14963333 10.1126/science.1089910

[34] Menon V. Large-scale brain networks and psychopathology: a unifying triple network model. Trends in Cognitive Sciences. 2011;15(10):483–506. doi: 10.1016/j.tics.2011.08.003 PubMed PMID: ISI:000295662800008. 21908230 10.1016/j.tics.2011.08.003

[35] Menon V, Uddin LQ. Saliency, switching, attention and control: a network model of insula function. Brain Structure & Function. 2010;214(5–6):655–67. doi: 10.1007/s00429-010-0262-0 PubMed PMID: WOS:000278898300021. 20512370 10.1007/s00429-010-0262-0 PMC2899886

[36] Craig AD. How do you feel—now? The anterior insula and human awareness. Nat Rev Neurosci. 2009;10(1):59–70. doi: 10.1038/nrn2555 PubMed PMID: WOS:000261934500015. 19096369 10.1038/nrn2555

[37] Rescorla RA, Wagner AW. A theory of Pavlovian conditioning: Variations in the effectiveness of reinforcement and nonreinforcement In: Black AH, Prokasy WF, editors. Classical Conditioning II: Current Research and Theory: Appleton-Century-Crofts; 1972 p. 64–99.

[38] Etkin A, Buchel C, Gross JJ. The neural bases of emotion regulation. Nat Rev Neurosci. 2015;16(11):693-+. doi: 10.1038/nrn4044 PubMed PMID: WOS:000363273000010. 26481098 10.1038/nrn4044

[39] Cooke M, Green P, Josifovski L, Vizinho A. Robust automatic speech recognition with missing and unreliable acoustic data. Speech Communication. 2001;34(3):267–85. doi: 10.1016/s0167-6393(00)00034-0 PubMed PMID: WOS:000168348700003.

[40] Rabiner LR. A tutorial on hidden Markov-models and selected applications in speech recognition. Proceedings of the IEEE. 1989;77(2):257–86. doi: 10.1109/5.18626 PubMed PMID: WOS:A1989U374600002.

[41] Anderson JR, Betts S, Ferris JL, Fincham JM. Neural imaging to track mental states while using an intelligent tutoring system. Proceedings of the National Academy of Sciences. 2010;107(15):7018–23. doi: 10.1073/pnas.1000942107 20335536 10.1073/pnas.1000942107 PMC2872451

[42] Anderson JR, Betts S, Ferris JL, Fincham JM. Tracking children's mental states while solving algebra equations. Human Brain Mapping. 2012;33(11):2650–65. doi: 10.1002/hbm.21391 21932262 10.1002/hbm.21391 PMC6870520

[43] Anderson JR, Fincham JM, Schneider DW, Yang J. Using brain imaging to track problem solving in a complex state space. NeuroImage. 2012;60(1):633–43. doi: 10.1016/j.neuroimage.2011.12.025 22209783 10.1016/j.neuroimage.2011.12.025 PMC3288582

[44] Borst JP, Anderson JR. The discovery of processing stages: Analyzing EEG data with hidden semi-Markov models. Neuroimage. 2015;108:60–73. doi: 10.1016/j.neuroimage.2014.12.029 PubMed PMID: WOS:000349618600006. 25534112 10.1016/j.neuroimage.2014.12.029

[45] Kaufman J, Birmaher B, Brent D, Rao UMA, Flynn C, Moreci P, et al Schedule for Affective Disorders and Schizophrenia for School-Age Children-Present and Lifetime Version (K-SADS-PL): Initial Reliability and Validity Data. Journal of the American Academy of Child & Adolescent Psychiatry. 1997;36(7):980–8. 9204677 10.1097/00004583-199707000-00021

[46] Cox RW. AFNI: Software for analysis and visualization of functional magnetic resonance neuroimages. Computers and Biomedical Research. 1996;29(3):162–73. doi: 10.1006/cbmr.1996.0014 PubMed PMID: WOS:A1996UV56700002. 8812068 10.1006/cbmr.1996.0014

[47] Smith SM, Jenkinson M, Woolrich MW, Beckmann CF, Behrens TEJ, Johansen-Berg H, et al Advances in functional and structural MR image analysis and implementation as FSL. NeuroImage. 2004;23:S208–S19. doi: 10.1016/j.neuroimage.2004.07.051 PubMed PMID: WOS:000225374100020. 15501092 10.1016/j.neuroimage.2004.07.051

[48] Kelly RE, Alexopoulos GS, Wang ZS, Gunning FM, Murphy CF, Morimoto SS, et al Visual inspection of independent components: Defining a procedure for artifact removal from fMRI data. Journal of Neuroscience Methods. 2010;189(2):233–45. doi: 10.1016/j.jneumeth.2010.03.028 PubMed PMID: WOS:000279088700012. 20381530 10.1016/j.jneumeth.2010.03.028 PMC3299198

[49] Tohka J, Foerde K, Aron AR, Tom SM, Toga AW, Poldrack RA. Automatic independent component labeling for artifact removal in fMRI. NeuroImage. 2008;39(3):1227–45. doi: 10.1016/j.neuroimage.2007.10.013 PubMed PMID: WOS:000252691800029. 18042495 10.1016/j.neuroimage.2007.10.013 PMC2374836

[50] Zeng WM, Qiu AQ, Chodkowski B, Pekar JJ. Spatial and temporal reproducibility-based ranking of the independent components of BOLD fMRI data. NeuroImage. 2009;46(4):1041–54. doi: 10.1016/j.neuroimage.2009.02.048 PubMed PMID: WOS:000266975600018. 19286465 10.1016/j.neuroimage.2009.02.048 PMC2746867

[51] Murphy K. The Bayes Net Toolbox for Matlab. Computing Science and Statistics. 2001.

[52] Baum LE, Petrie T, Soules G, Weiss N. A maximization technique occurring in the statistical analysis of probabilistic functions of Markov chains. Ann Math Statist. 1970;41(1):164–71.

[53] Akaike H. New look at statistical-model identification. Ieee Transactions on Automatic Control. 1974;AC19(6):716–23. doi: 10.1109/tac.1974.1100705 PubMed PMID: WOS:A1974U921700011.

[54] Anderson DR, Burnham KP. Avoiding pitfalls when using information-theoretic methods. Journal of Wildlife Management. 2002;66(3):912–8. doi: 10.2307/3803155 PubMed PMID: WOS:000177475600033.

[55] Hannan EJ, Quinn BG. Determination of the order of an autogregression. J R Stat Soc Ser B-Methodol. 1979;41(2):190–5. PubMed PMID: WOS:A1979HH71100005.

[56] Schwarz G. Estimating dimension of a model. Annals of Statistics. 1978;6(2):461–4. doi: 10.1214/aos/1176344136 PubMed PMID: WOS:A1978EQ63300014.

[57] Viterbi AJ. Error bounds for convolutional codes and an asymptotically optimum decoding algorithm. Ieee Transactions on Information Theory. 1967;13(2):260-+. doi: 10.1109/tit.1967.1054010 PubMed PMID: WOS:A19679497100016.

[58] Posner MI, Snyder CR, Davidson BJ. Attention and the detection of signals. Journal of Experimental Psychology. 1980;109(2):160–74. doi: 10.1037//0096-3445.109.2.160 PubMed PMID: MEDLINE:7381367. 7381367

[59] Grinband J, Savitskaya J, Wager TD, Teichert T, Ferrera VP, Hirsch J. The dorsal medial frontal cortex is sensitive to time on task, not response conflict or error likelihood. Neuroimage. 2011;57(2):303–11. doi: 10.1016/j.neuroimage.2010.12.027 PubMed PMID: WOS:000291960200001. 21168515 10.1016/j.neuroimage.2010.12.027 PMC3114292

[60] Neta M, Schlaggar BL, Petersen SE. Separable responses to error, ambiguity, and reaction time in cingulo-opercular task control regions. Neuroimage. 2014;99:59–68. doi: 10.1016/j.neuroimage.2014.05.053 PubMed PMID: WOS:000339860000007. 24887509 10.1016/j.neuroimage.2014.05.053 PMC4148211

[61] James TW, Culham J, Humphrey GK, Milner AD, Goodale MA. Ventral occipital lesions impair object recognition but not object-directed grasping: an fMRI study. Brain. 2003;126:2463–75. doi: 10.1093/brain/awg248 PubMed PMID: WOS:000186144800012. 14506065 10.1093/brain/awg248

[62] Macko KA, Jarvis CD, Kennedy C, Miyaoka M, Shinohara M, Sokoloff L, et al Mapping the primate visual-system with 2-C-14 labeled deoxyglucose. Science. 1982;218(4570):394–7. doi: 10.1126/science.7123241 PubMed PMID: WOS:A1982PL04000038. 7123241 10.1126/science.7123241

[63] Breiter HC, Etcoff NL, Whalen PJ, Kennedy WA, Rauch SL, Buckner RL, et al Response and habituation of the human amygdala during visual processing of facial expression. Neuron. 1996;17(5):875–87. doi: 10.1016/s0896-6273(00)80219-6 PubMed PMID: WOS:A1996VV15100010. 8938120 10.1016/s0896-6273(00)80219-6

[64] Cservenka A, Stroup ML, Etkin A, Nagel BJ. The effects of age, sex, and hormones on emotional conflict-related brain response during adolescence. Brain and Cognition. 2015;99:135–50. doi: 10.1016/j.bandc.2015.06.002 PubMed PMID: WOS:000361160200015. 26175008 10.1016/j.bandc.2015.06.002 PMC4555000

