# Supplementary figures and images for: Implicit emotion regulation in adolescent girls: An exploratory investigation of Hidden Markov Modeling and its neural correlates
Source: PLoS One. 2018 Feb 28;13(2):e0192318. doi: 10.1371/journal.pone.0192318 (PMC5830311; doi:10.1371/journal.pone.0192318)

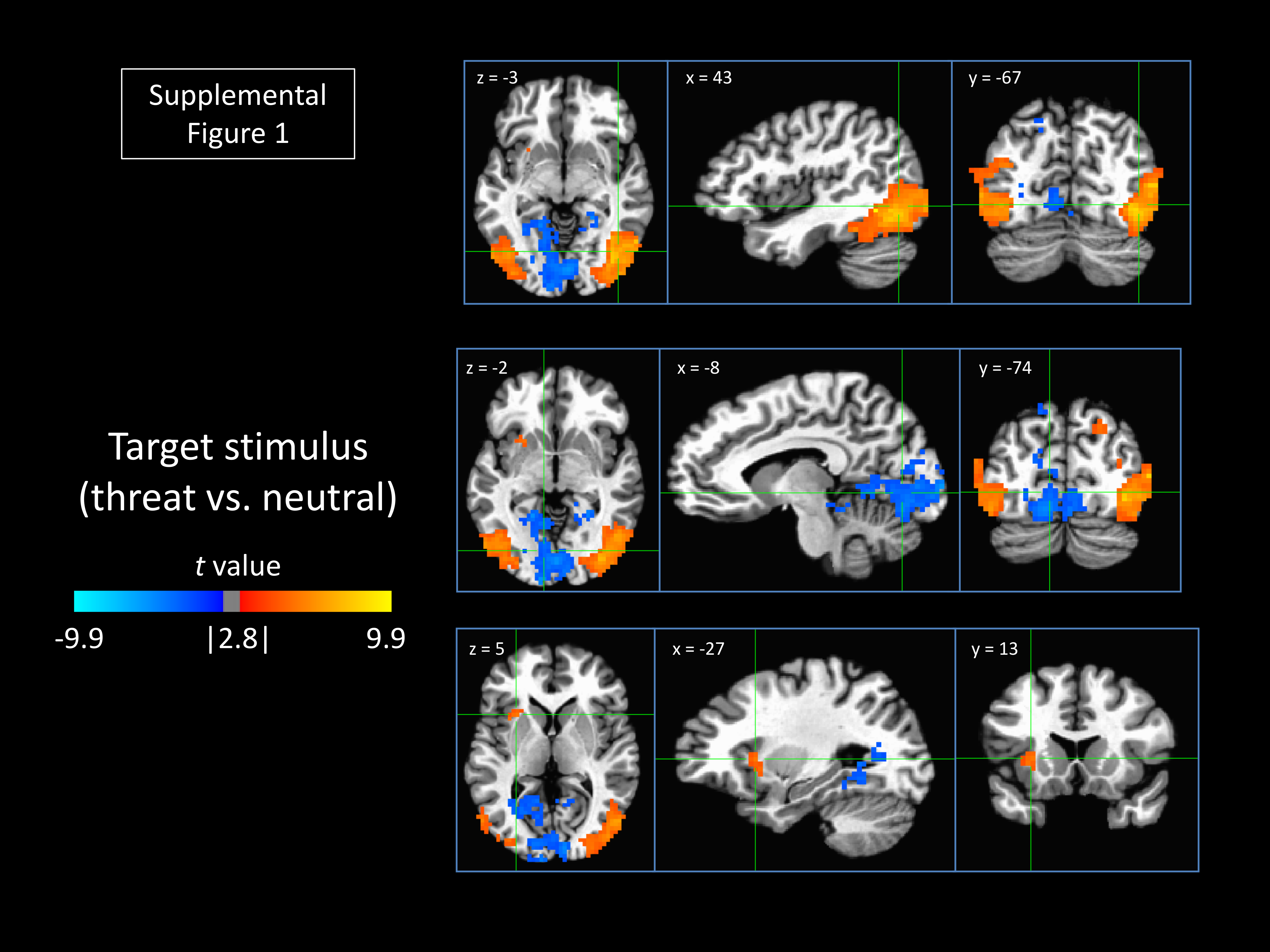

Supplement: S1 Fig — Shown are areas that were differentially responsive to the actual presence of threatening versus neutral target stimuli (not the detection of the stimuli). Positive values (orange) indicate that a region was activated in response to threatening target stimuli and/or deactivated by neutral target stimuli. Likewise, the negative values (blue) follow the inverse of this relationship. (TIF) [file pone.0192318.s001.tif]

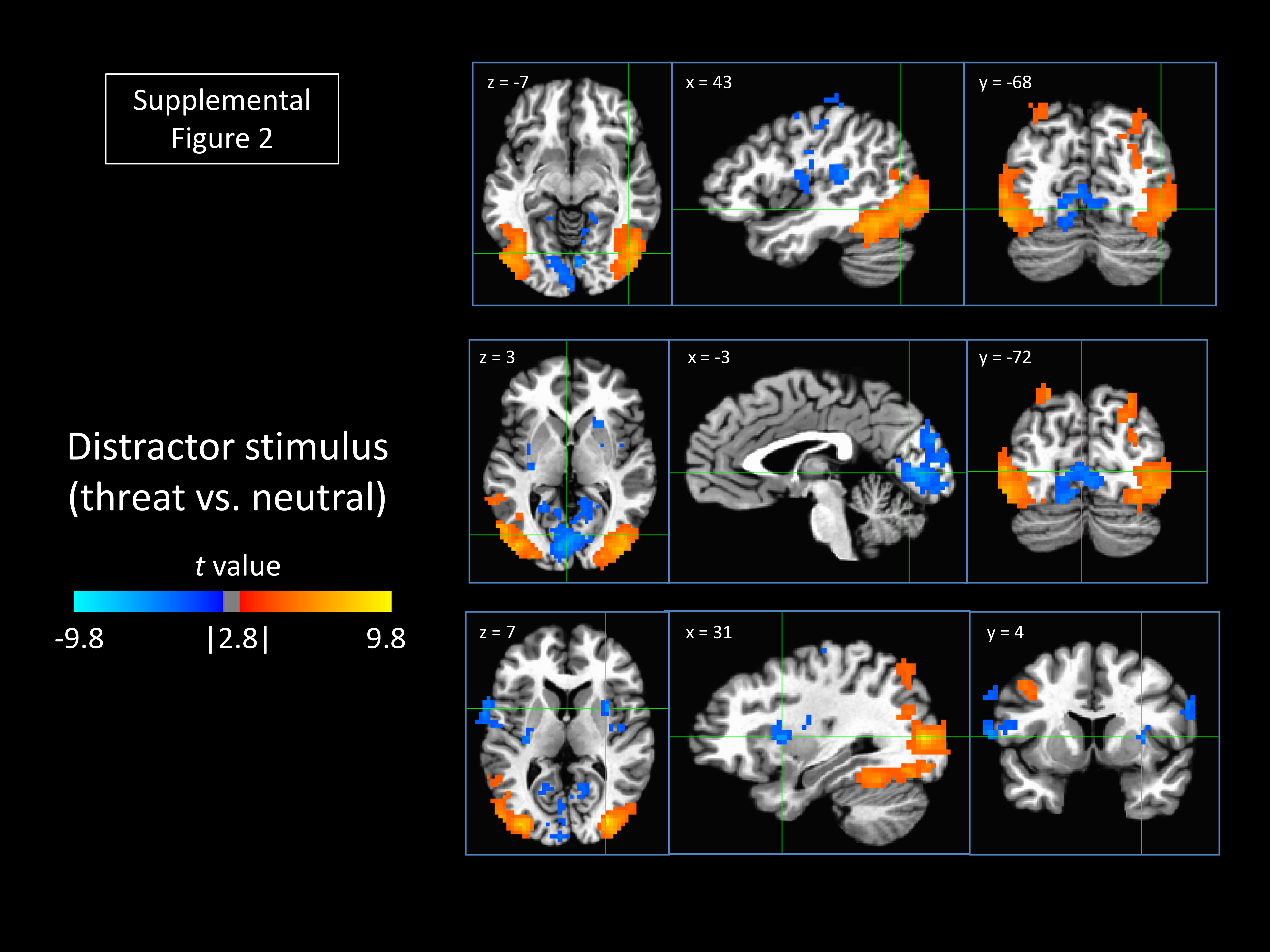

Supplement: S2 Fig — Shown are areas that were differentially responsive to the actual presence of threatening versus neutral distractor stimuli (not the detection of the stimuli). Positive values (orange) indicate that a region was activated in response to threatening target stimuli and/or deactivated by neutral target stimuli. Likewise, the negative values (blue) follow the inverse of this relationship. (TIF) [file pone.0192318.s002.tif]
