# Supplementary material for: Implicit emotion regulation in adolescent girls: An exploratory investigation of Hidden Markov Modeling and its neural correlates
Source: PLoS One. 2018 Feb 28;13(2):e0192318. doi: 10.1371/journal.pone.0192318 (PMC5830311; doi:10.1371/journal.pone.0192318)
Supplement: S1 Table — (DOCX) [file pone.0192318.s003.docx]

Supplemental Table 1 – Contrast-based measures of normative task performance.

|  |  | |  |
| --- | --- | --- | --- |
|  | *t* | *p* | *mean (SD) (ms)* |
| Valence (threat/threat vs. neutral/neutral) | -2.23 | 0.036* | -38 (68) |
| Conflict bias | 2.04 | 0.053 | 5.0 (68) |
| Fear distractor conflict bias | 4.23 | 0.0003* | 65 (70) |
| Fear target conflict bias | -2.20 | 0.038* | -31 (70) |
| Adapation to conflict | 2.07 | 0.050* | 15 (66) |
| Adapation following fear distractor conflict | 4.34 | 0.0002* | 30 (32) |
| Adaptation following fear target conflict | -1.48 | 0.152 | -44 (106) |
| Slow emotional interference | -3.45 | 0.002* | -38 (81) |
| Post error slowing | -1.97 | 0.061 | 49 (100) |
