# Supplementary material for: Implicit emotion regulation in adolescent girls: An exploratory investigation of Hidden Markov Modeling and its neural correlates
Source: PLoS One. 2018 Feb 28;13(2):e0192318. doi: 10.1371/journal.pone.0192318 (PMC5830311; doi:10.1371/journal.pone.0192318)
Supplement: S2 Table — (DOCX) [file pone.0192318.s004.docx]

Supplemental Table 2 – Contrast-based measures, excluding incorrect trials and trials subsequent to incorrect trials

|  |  | |  | |
| --- | --- | --- | --- | --- |
|  | *t* | *p* |  |  |
| Valence (threat/threat vs. neutral/neutral) | -2.08 | 0.049* |  |  |
| Conflict bias | 1.20 | 0.243 |  |  |
| Fear distractor conflict bias | 3.36 | 0.001* |  |  |
| Fear target conflict bias | -2.43 | 0.023* |  |  |
| Adapation to conflict | 1.95 | 0.063 |  |  |
| Adapation following fear distractor conflict | 4.65 | 0.001* |  |  |
| Adaptation following fear target conflict | -1.58 | 0.129 |  |  |
| Slow emotional interference | -3.94 | 0.001* |  |  |
