# Supplementary material for: Implicit emotion regulation in adolescent girls: An exploratory investigation of Hidden Markov Modeling and its neural correlates
Source: PLoS One. 2018 Feb 28;13(2):e0192318. doi: 10.1371/journal.pone.0192318 (PMC5830311; doi:10.1371/journal.pone.0192318)
Supplement: S3 Table — (DOCX) [file pone.0192318.s005.docx]

Supplemental Table 3. Optimal model parameters.

For binary nodes the parameters are listed for one configuration only. The parameters for the other configuration are the complement of the parameters listed.

|  |  | Target stimulus | |  | |
| --- | --- | --- | --- | --- | --- |
|  |  | Neutral | Threatening |  |  |
| Task effort | High | 0.830 | 0.599 | Target Detector Neutral | |
|  | Low | 0.835 | 0.698 |  |  |
|  |  |  |  |  |  |
|  |  | Distractor stimulus | |  |  |
|  |  | Neutral | Threatening |  |  |
| Threat evaluation | High | 0.2414 | 0.275 | Distractor Detector Neutral | |
|  | Low | 0.995 | 0.537 |  |  |
|  |  |  |  |  |  |
|  |  | Distractor detector | |  |  |
|  |  | Neutral | Threatening |  |  |
| Target detector | Neutral | 0.108 | 0.738 | Task Effort High (subsequent trial) | |
|  | Threatening | 0.681 | 0.222 |  |  |
|  |  |  |  |  |  |
|  |  | Distractor detector | |  |  |
|  |  | Neutral | Threatening |  |  |
| Target detector | Neutral | 0.496 | 0.833 | Threat Evaluation High (subsequent trial) | |
|  | Threatening | 0.602 | 0.944 |  |  |
|  |  |  |  |  |  |
|  |  | Distractor detector | |  |  |
|  |  | Neutral | Threatening |  |  |
| Target detector | Neutral | 0.895 | 0.06 | Fast response | |
|  | Threatening | 0.732 | 0.446 |  |  |
|  |  |  |  |  |  |
|  |  | Distractor detector | |  |  |
|  |  | Neutral | Threatening |  |  |
| Target detector | Neutral | 0.095 | 0.831 | Slow response | |
|  | Threatening | 0.032 | 0.164 |  |  |
|  |  |  |  |  |  |
|  |  | Distractor detector | |  |  |
|  |  | Neutral | Threatening |  |  |
| Target detector | Neutral | 0.01 | 0.11 | Incorrect response | |
|  | Threatening | 0.236 | 0.39 |  |  |
